# Comprehensive analysis of trihelix genes and their expression under biotic and abiotic stresses in *Populus trichocarpa*

**DOI:** 10.1038/srep36274

**Published:** 2016-10-26

**Authors:** Zhanchao Wang, Quangang Liu, Hanzeng Wang, Haizhen Zhang, Xuemei Xu, Chenghao Li, Chuanping Yang

**Affiliations:** 1 State Key Laboratory of Tree Genetics and Breeding, Northeast Forestry University, 26 Hexing Road, Harbin 150040, China; 2 Library of Northeast Forestry University, 26 Hexing Road, Harbin 150040, China

## Abstract

Trihelix genes play important roles in plant growth and development and responses to biotic and abiotic stresses. Here, we identified 56 full-length trihelix genes in *Populus trichocarpa* and classified them into five groups. Most genes within a given group had similar gene structures and conserved motifs. The trihelix genes were unequally distributed across 19 different linkage groups. Fifteen paralogous pairs were identified, 14 of which have undergone segmental duplication events. Promoter *cis*-element analysis indicated that most trihelix genes contain stress- or phytohormone-related *cis*-elements. The expression profiles of the trihelix genes suggest that they are primarily expressed in leaves and roots. Quantitative real-time reverse transcription polymerase chain reaction analysis indicated that members of the trihelix gene family are significantly induced in response to osmotic, abscisic acid, salicylic acid, methyl jasmonate and pathogen infection. *PtrGT10* was identified as a target gene of miR172d, which is involved in the osmotic response. Repression of *PtrGT10* could increase reactive oxygen species scavenging ability and decrease cell death. This study provides novel insights into the phylogenetic relationships and functions of the *P. trichocarpa* trihelix genes, which will aid future functional studies investigating the divergent roles of trihelix genes belonging to other species.

Trihelix proteins comprise one of the first families of transcription factors discovered in plants and are classified as GT factors due to their binding specificity for GT elements^1^. GT elements are highly degenerated, and the deduced consensus core sequence is 5′-G-Pu-(T/A)-A-A-(T/A)3′^2^. To date, 30 trihelix proteins have been identified in *Arabidopsis*, and 31 have been identified in rice. The *Arabidopsis* trihelix proteins are grouped into five classes, namely, GT-1, GT-2, SH-4, GTγ and SIP1. Each class is named after the corresponding founding member^3^. The DNA-binding domain of GT factors features a typical trihelix (helix-loop-helix-loop-helix) structure. Although each trihelix subfamily has at least one trihelix structure, small differences still exist. For example, in the GT-2 and GTγ subfamilies, a conserved tryptophan is replaced by phenylalanine (F), while in the SIP1 subfamily, it is replaced by isoleucine (I).

Trihelix transcription factors play important roles in the regulation of developmental processes involving flowers^4^, trichomes, stomata, seed abscission layers and late embryogenesis and in responses to biotic and abiotic stresses^5^,^6^,^7^ or to treatments with phytohormones such as abscisic acid (ABA) or salicylic acid (SA)^8^. The *Arabidopsis* gene *PETAL LOSS (PTL*), which belongs to the GT-2 group, regulates petal and sepal growth as well as sepal fusion^9^. A pair of *Arabidopsis* genes in the GT-1 clade, *GT-3a* and *GT-3b*, have been shown to respond to salt and pathogen stress^10^. A GT-factor in rice, *OsRML1*, was reported to be induced in response to the rice pathogen *Magnaporthe grisea*^11^. The *Arabidopsis* GT-2 Like 1 (*ATGTL1*) gene negatively regulates water use efficiency by modulating stomatal density, with mutations leading to increased plant tolerance to drought stress^12^. Two genes encoding putative GT-2-type proteins in soybean (*GmGT-2A* and *GmGT-2B*) were shown to stimulate enhanced tolerance to salt, drought and freezing stresses upon overexpression in transgenic *Arabidopsis* plants^13^.

Poplar (*Populus trichocarpa*) trees are perennial woody deciduous plants with significant commercial and ecological value^14^. Studies of poplars have become prevalent in recent years because of their economic significance for pulp and biofuel production. Poplars are frequently threatened by environmental stresses such as drought, cold and salt. Although trihelix genes have been investigated in *Arabidopsis*, rice, and soybean, studies in poplar are still limited. To date, only one trihelix gene has been studied functionally in poplars, and the results suggest that trihelix genes may play important roles in the drought response^15^. Therefore, there is a need for a comprehensive analysis of this protein family in poplars.

In this study, we report the genome-wide identification, phylogenetic analysis, gene structure and promoter *cis*-elements of 56 novel trihelix genes in *P. trichocarpa*. We measured gene expression during biotic and abiotic stresses and phytohormone treatments. We also identified a target gene of miR172d, *PtrGT10*, which may function during osmotic stress. Our preliminary results provide novel insights into the roles of trihelix genes in the poplar’s responses to biotic and abiotic stresses and phytohormone treatments that will aid future studies.

## Results

### Identification of trihelix genes in *Populus*

The Phytozome V10.3 and NCBI databases were used to identify trihelix genes in the *P. trichocarpa* genome. In total, 80 candidate trihelix genes were identified, all of which were examined for the presence of the trihelix domain (PF13837, SM00717) using the Pfam 28.0 and SMART databases. These genes were then compared with the trihelix gene family in PlnTFDB V3.0, which contained 78 candidate trihelix genes. By removing repetitive and redundant genes, we finally identified 56 putative trihelix genes in *P. trichocarpa*. The number of trihelix genes in *P. trichocarpa* is much higher than those in *Arabidopsis* and rice (30 and 31, respectively)^3^. According to the nomenclature of trihelix genes in *Arabidopsis*^16^ and soybean^13^, we named the 56 *Populus* trihelix genes *PtrGT1* to *PtrGT56*. Of the 56 trihelix genes, 39 had only one gene product, while the remaining 17 genes (30%) had multiple gene products, which could be attributed to alternative splicing according to the Phytozome V10.3 database. This percentage was similar to that of the C2H2 gene family (26%) in *P. trichocarpa*^17^. Among these 17 genes, 15 encoded two possible cDNAs, while *PtrGT24* and *PtrGT37* each encoded three possible transcripts (Supplementary Table S1).

The proteins encoded by these 56 trihelix genes ranged from 251 to 994 amino acids (aa) in length, with an average length of 453 aa. The trihelix protein sequences showed large variations in isoelectric point (pI) values (ranging from 4.29 to 10.36) and molecular weight (ranging from 51.475 kDa to 111.294 kDa). Localization predictions made with the Wolf PSORT database classified 50 of the trihelix proteins as nuclear proteins, five as cytoplasmic or chloroplast proteins and only one as a peroxisomal protein (*PtrGT53*). Additional information about the 56 trihelix genes in *P. trichocarpa* is provided in Supplementary Table S2 and Supplementary Data S1.

### Phylogenetic analysis and gene structure of the trihelix gene family

**Figure 1:**
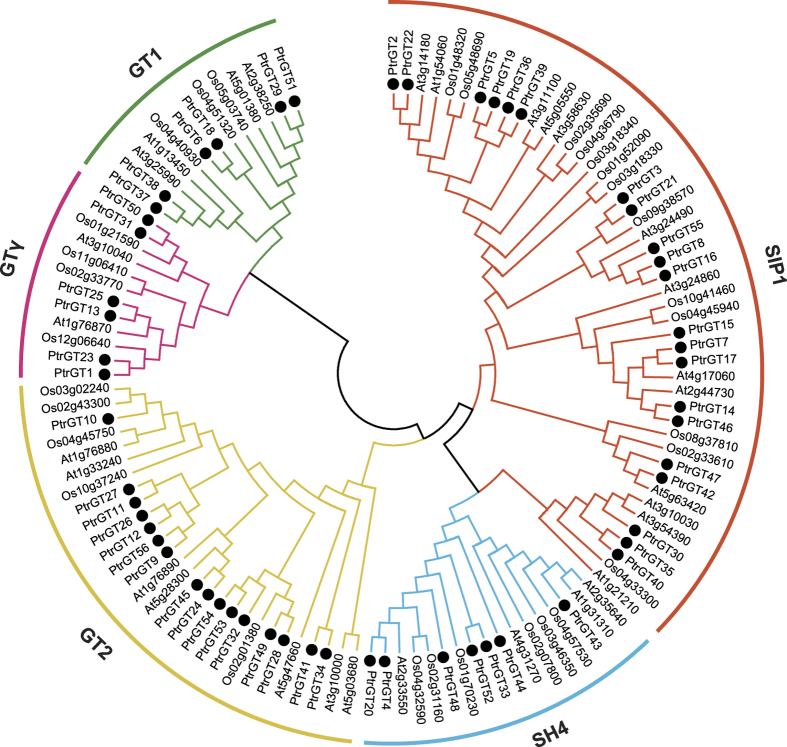
Phylogenetic tree of trihelix proteins from *Populus, Arabidopsis* and rice. Full-length amino acid sequences were aligned by ClustalX 1.83, and the NJ tree was constructed by MEGA5.0 with 1000 bootstrap replicates. Each trihelix subfamily is indicated in a specific color. *Populus* trihelix proteins are marked with solid dots.

An unrooted phylogenetic tree was constructed using the full-length protein sequences of *P. trichocarpa, Arabidopsis* and rice (Fig. 1). The *P. trichocarpa* trihelix genes grouped into five subgroups (GT-1, GTγ, GT-2, SH4, and SIP1) according to the previous study in *Arabidopsis*^18^, with six GT-1, six GTγ, 16 GT-2, seven SH4, and 21 SIP1 members. Based on this analysis, 15 pairs of paralogous genes were identified, all of which had strong bootstrap support (>90%) (Table 1). A multiple sequence alignment is shown in Supplementary Fig. S1 and shows similar characteristics as previously observed^3^.

**Figure 2:**
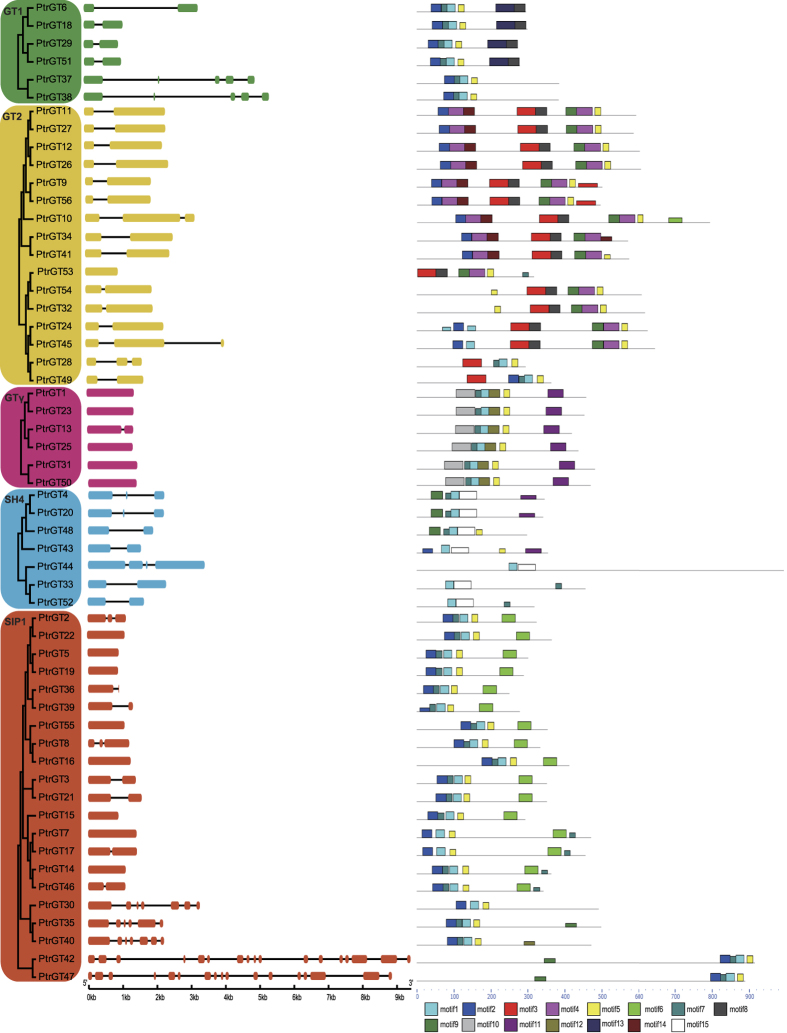
Phylogenetic relationships, gene structures and motif compositions of *Populus* trihelix genes. (**a**) An unrooted phylogenetic tree generated with the MEGA5.0 program using the full-length amino acid sequences with 1000 bootstrap replicates. The five subfamilies are marked with different colors. (**b**) Exon/intron structures of *Populus* trihelix genes. Exons and introns of each subfamily are represented by colored boxes and black lines, respectively. (**c**) Schematic representation of the conserved motifs in *Populus* trihelix proteins by MEME. Each colored box represents a motif, and black lines represent non-conserved sequences.

Figure 2b shows that the most closely related trihelix members within the same subfamilies shared similar gene structures in terms of intron numbers or exon lengths. The similarity in gene structures was consistent with the phylogenetic analysis. Interestingly, most genes had only one or two exons, except *PtrGT30, 35, 40, 42* and *47* in the SIP1 subfamily.

Putative conserved motifs predicted by MEME show the diversity of the *P. trichocarpa* trihelix genes (Fig. 2c). In this prediction, 15 distinct motifs were identified. The best possible match and domain are shown in Supplementary Table S3. As expected, most of the closely related members had common motif compositions, suggesting functional similarities among the trihelix proteins in the same subgroup.

### Chromosomal locations and duplications analysis

**Figure 3:**
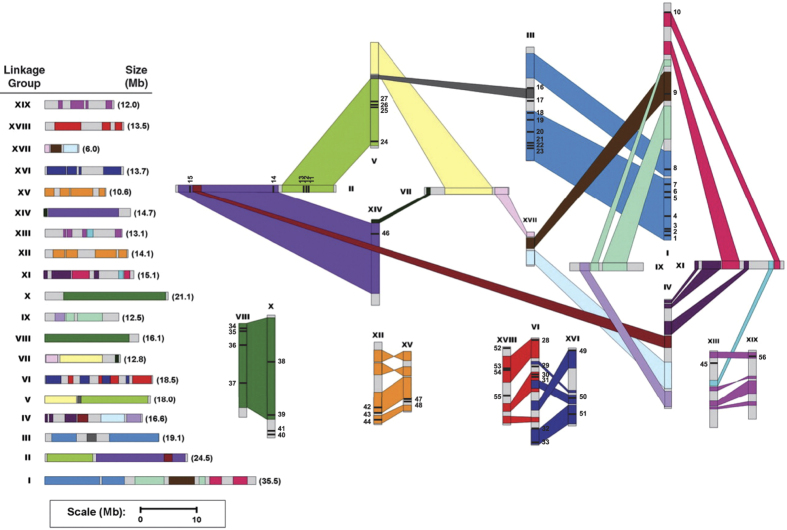
Chromosomal locations of *Populus* trihelix genes. Segmental duplicated homologous blocks are indicated by the same color. The scale represents mega bases (Mb). The LG numbers are indicated above each bar.

Trihelix genes were physically mapped on 19 linkage groups (LGs). LGI contains 10 genes, which was the highest number, followed by eight genes on LGIII. In contrast, no genes were located on LGIV, LGVII, LGIX, LGXI or LGXVII (Fig. 3).

According to a previous study, at least three rounds of genome-wide duplications occurred in the *Populus* genome, followed by multiple segmental duplications, tandem duplications and transposition events^19^. In our study, we mapped the *P. trichocarpa* trihelix genes to the duplicated blocks identified in the previous study^19^. Forty-four genes were located in duplicated regions, and 30 of those were present in both duplicates, while the others were only present in one of the blocks (Fig. 3). By contrast, 12 trihelix genes were located outside the duplicated blocks, suggesting that the loss of some genes is caused by dynamic changes after segmental duplication.

### Promoter *cis*-element analysis

The promoter regions of *P. trichocarpa* trihelix genes contained numerous abiotic stress response- and phytohormone-related *cis*-elements (Supplementary Table S4). These trihelix promoters also contained *cis*-elements related to biotic stresses, such as EIRE and ELI-box3. Most trihelix promoters (46) contained HSE elements, followed by G-Box and TC-rich repeats (43 genes). Of all of the genes, the *PtrGT14* and *PtrGT26* promoters contained the most *cis*-elements (11), while *PtrGT4* only had two *cis*-elements (Supplementary Table S5).

### Gene Ontology (GO) annotation analysis

**Figure 4:**
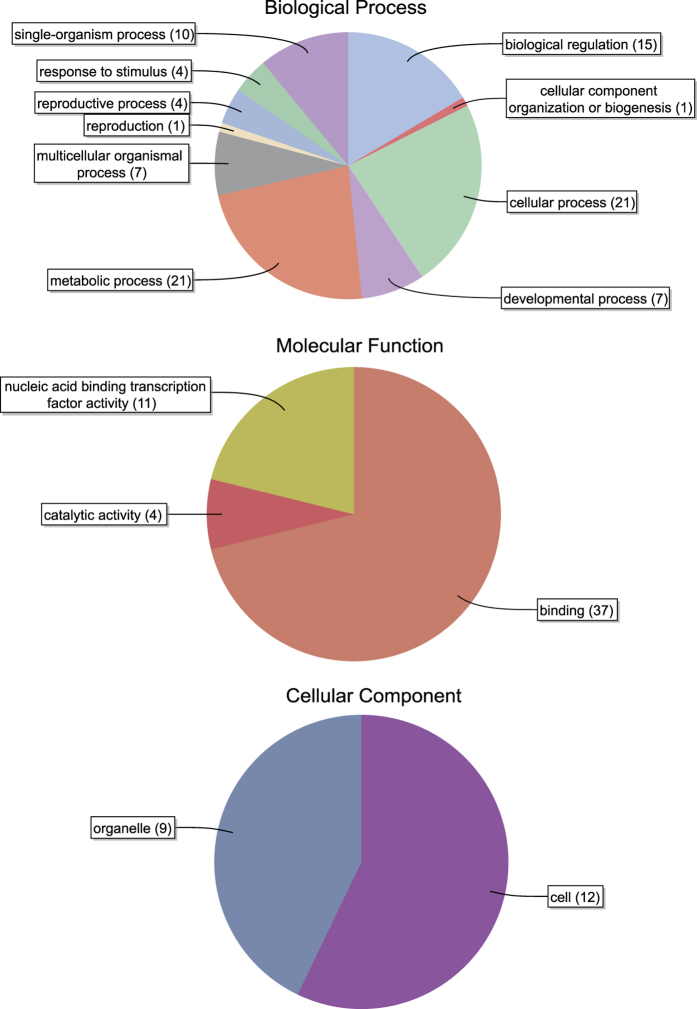
Gene Ontology (GO) results for *Populus* trihelix proteins.

GO annotation analysis of the 56 trihelix genes associated them with different biological processes. Of the 10 GO terms associated with trihelix genes, metabolic process and cellular process were the categories with the largest numbers of genes (21), followed by biological regulation (15). *PtrGT31* and *PtrGT50* are related to stress responses (Supplementary Table S6). Only one gene was predicted to function in reproduction and cellular component organization or biogenesis. As for genes in the molecular function category, most were annotated with binding ability (37), while only four genes were annotated to have catalytic activity. Under the cellular component term, 12 genes were located in the cell part, while nine genes were associated with organelles (Fig. 4).

### Expression profiles of trihelix genes in *P. trichocarpa*

**Figure 5:**
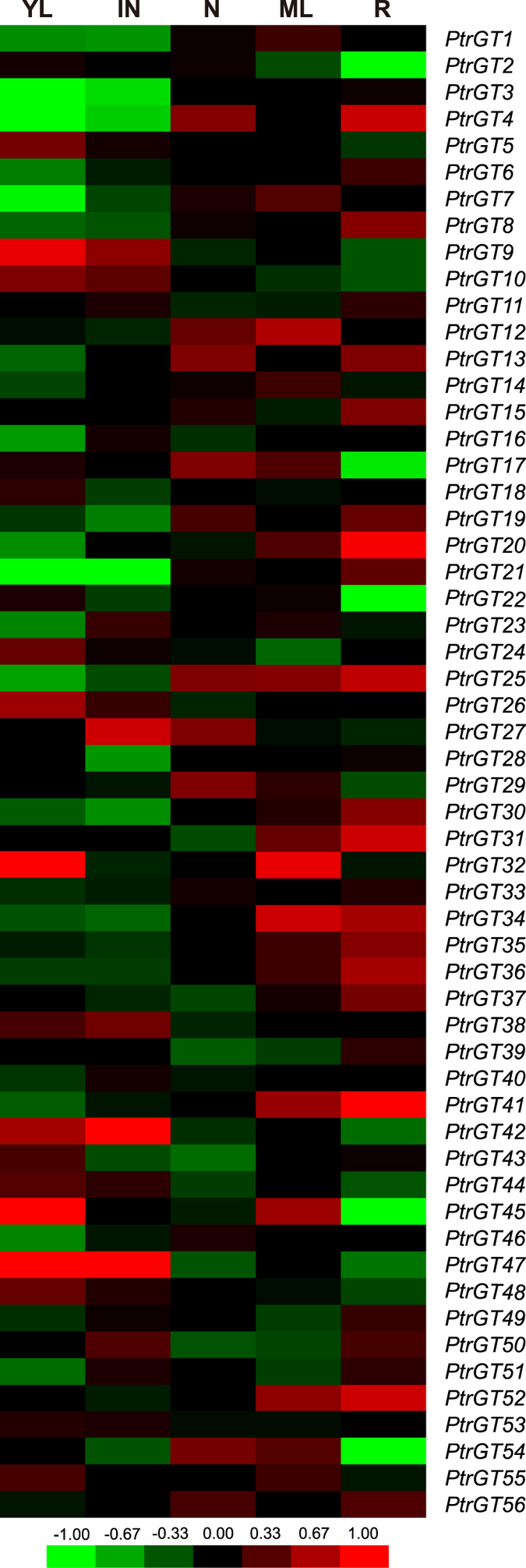
ExImage analysis of Populus trihelix genes in different tissues. YL, IN, N, ML and R refer to young leaves, internodes, nodes, mature leaves and roots, respectively. The exImage was visualized by the exImage tool in the PopGenIE v3.0 database. Red and green indicate high and low levels of transcript abundances, respectively.

The exImage tool in the PopGenIE v3.0 database compiled gene expression patterns in different *Populus* tissues using public microarray datasets (accession number: GSE6422)^20^. In Fig. 5, 20 trihelix genes were co-enriched in young leaves, inter nodes and mature leaves, 24 trihelix genes were enriched in nodes, 29 were enriched in roots, and 15 genes were co-expressed in internodes and nodes. Similar numbers of genes were expressed in the other three tissues (nine in roots and 10 in both young leaves and mature leaves).

Trihelix genes have previously been shown to play crucial roles in the drought stress response^13^. The expression profiles of trihelix genes in response to drought stress were thus investigated using the publicly available explot data in the exPlot tool of the PopGenIE v3.0 database. As shown in Supplementary Figs S2 and 23 trihelix genes were upregulated in leaves and roots, while 16 genes were downregulated in leaves, and 27 genes were downregulated in roots. Of these genes responding to drought, 11 were upregulated in both leaves and roots, while seven were downregulated in both leaves and roots.

### Examination of trihelix gene expression by qRT-PCR

**Figure 6:**
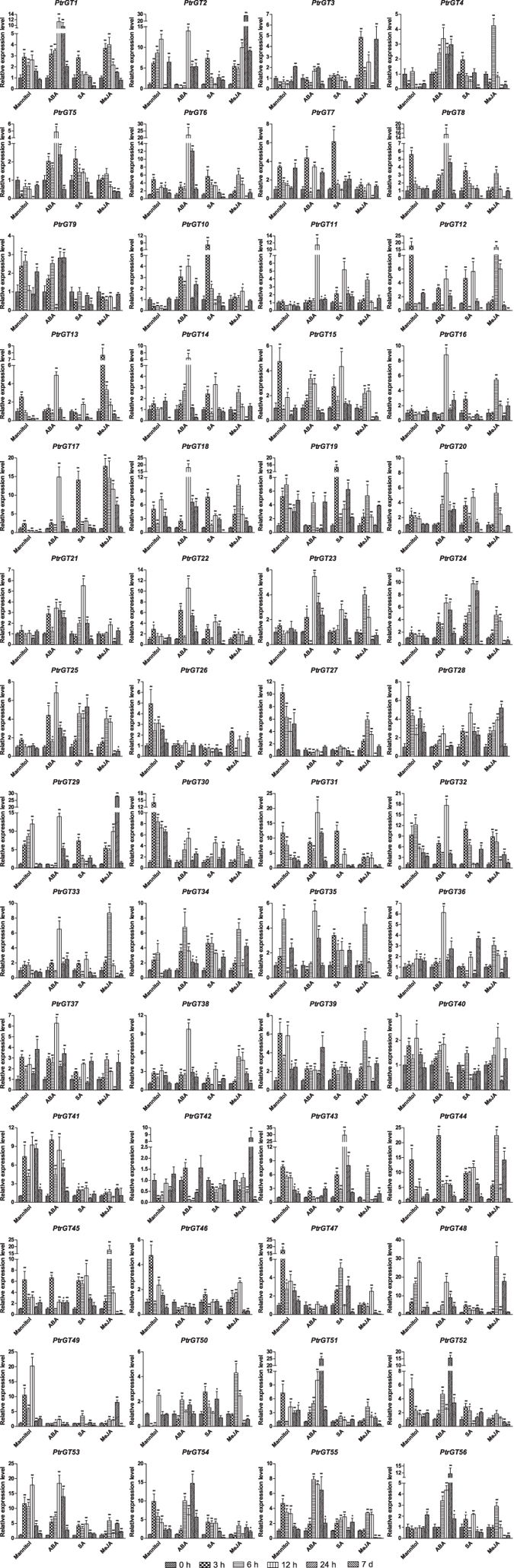
Expression analysis of trihelix genes in leaves under osmotic, ABA, SA and MeJA by qRT-PCR. The x-axis represents time after the onset of stress treatments. Error bars represent the standard deviations of three biological replicates. Asterisks indicate stress treatment groups that showed a significant difference in transcript abundance compared with the control group (*P < 0.05, **P < 0.01).

To measure individual gene expression of *P. trichocarpa* trihelix genes, qRT-PCR was conducted under osmotic stress or ABA, SA, methyl jasmonate (MeJA) or pathogen treatment for 0 h, 3 h, 6 h, 12 h, 24 h or 7 d (Fig. 6). To verify that the treatments were working well, four marker genes (*RD29A, RAB18, PR1* and *PR4*) were used as positive controls under these conditions. As expected, the expressions of these marker genes were all significantly induced after treatments (Supplementary Fig. S3). Genes up- or downregulated by more than 2.0-fold were considered significantly differentially expressed^21^. For osmotic stress, 42 trihelix genes were induced, eight trihelix genes were suppressed, and six trihelix genes showed no change. Notably, *PtrGT12, 27, 29, 30, 31, 32, 44, 47, 48, 49, 53* and *54* were significantly upregulated (>10.0-fold relative to the control). Moreover, *PtrGT2, 6, 19, 20, 25, 27, 28, 30, 40, 41, 43, 49* and *55* were upregulated at 3 h, 6 h, 12 h and 24 h in leaves, whereas only *PtrGT3, PtrGT5* and *PtrGT10* were downregulated at 3 h, 6 h, 12 h and 24 h. The expression trends were generally consistent with the exPlot analysis. Most trihelix genes were downregulated at day 7 of mannitol treatment, except for *PtrGT3, 10, 37, 42* and *56.*

Under ABA stress, 50 trihelix genes were upregulated, and five genes (*PtrGT26, 27, 42, 46* and *47*) were downregulated. Thirteen trihelix genes (*PtrGT1, 8, 17, 22, 31, 32, 41, 44, 51, 52, 53, 54* and *56*) were significantly upregulated at 3 h, 6 h, 12 h and 24 h, while only three genes (*PtrGT27, 46* and *47*) were downregulated. Under SA stress, 47 genes were upregulated, and eight genes were downregulated; six genes (*PtrGT17, 24, 31, 32, 43* and *44*) were significantly upregulated, whereas only *PtrGT9* was downregulated. Under MeJA stress, 49 trihelix genes were upregulated, and seven were downregulated, similar to the SA stress results. Only eight genes (*PtrGT2, 12, 17, 18, 29, 44, 45* and *48*) were significantly upregulated, while *PtrGT9* was significantly downregulated. Most trihelix genes were downregulated under ABA, SA and MeJA treatments, whereas *PtrGT9, 19, 39, 42*and *43* were upregulated in ABA treatment and *PtrGT36, 37* and *PtrGT3* were upregulated in SA and MeJA treatments, respectively.

**Figure 7:**
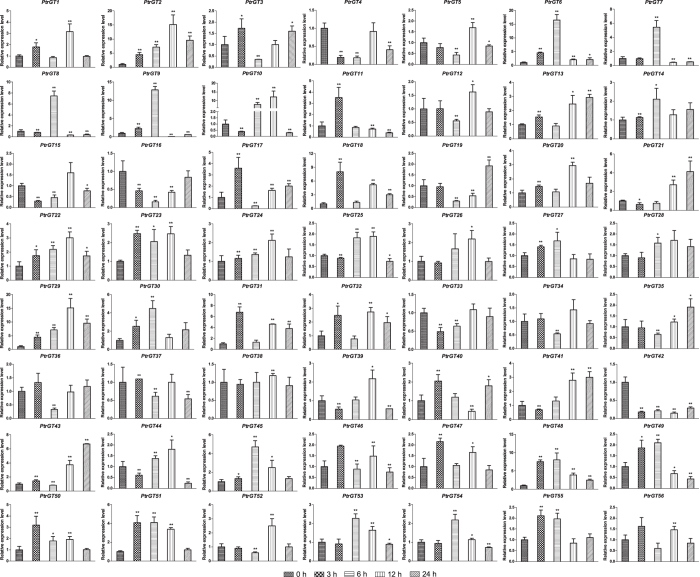
Expression analysis of trihelix genes in leaves under pathogen infection treatments by qRT-PCR. The x-axis represents time after the onset of stress treatments. Error bars represent the standard deviations of three biological replicates. Asterisks indicate stress treatment groups that showed a significant difference in transcript abundance compared with the control group (*P < 0.05, **P < 0.01).

According to a previous study, GT factors play a role in pathogen-induced *SCaM-4* gene expression in both soybean and *Arabidopsis*^10^. In our study, because some genes have *cis*-elements related to fungal infection, we also carried out qRT-PCR during *Alternaria alternate* (pathogen of leaf blight) infection (Fig. 7). We observed that 35 genes were upregulated, 15 were downregulated, and six showed no obvious change. *PtrGT2, 6, 9, 10* and *29* were significantly upregulated at all time points, whereas *PtrGT42* was downregulated at all time points.

### MicroRNA target sites and expression analysis

**Figure 8:**
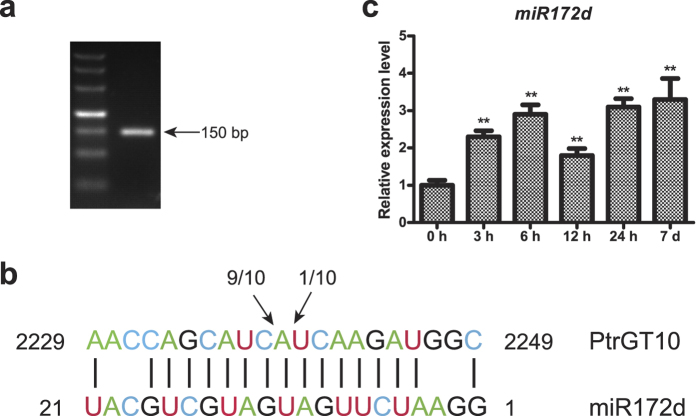
The cleavage site of *miR172d* on *PtrGT10*. (**a**) Agarose gel electrophoresis of the modified 5′RACE experiment on *PtrGT10*. (**b**) Alignment of the target site in *PtrGT10* with *miR172d*. The arrow and the numbers above the sequences indicate the cleavage site and the number of sequenced clones that revealed cleavage in that position, respectively. (**c**) The relative expression of *miR172d* under osmotic stress. The bars indicate standard deviation. The asterisks indicate statistically significant differences between the relative expression of control and treatment groups (*P < 0.05, **P < 0.01).

To study the relationship between trihelix genes and miRNAs, the psRNATarget online tool was used to find putative trihelix gene targets and found none. We then searched the flower bud target database during early stages (pollen at tetrad) obtained from our previous study using high-throughput sequencing. Here, we found a putative target of *miR172d*. Validation of miRNA cleavage was conducted using 5′ RLM RACE. Figure 8a shows the agarose gel electrophoresis of the 5′ RACE products of *PtrGT10*. The identified cleavage validation at the 10th base of *miR172d* is shown in Fig. 8b, while the original sequencing peaks were shown in Supplementary Fig. S4. These results demonstrate that *PtrGT10* is a target gene of *miR172d*.

We then measured *miR172d* expression levels by qRT-PCR. The results showed that *miR172d* was upregulated under osmotic stress (Fig. 8c), opposite to the observed pattern for of *PtrGT10* expression. This result is consistent with the typical relationship between miRNAs and their target genes.

### Generation of transiently transformed *P. ussuriensis* plants with repression of *PtrGT10*

*Agrobacterium*-mediated transient transformation systems are powerful tools for analyzing the function of genes and the generation of gene products^22^. In this study, two types of transiently transformed *P. ussuriensis* plants were generated, i.e., plants transform with *35S::PtrGT10SRDX* for dominant repressing *PtrGT10* (DR) and control (transformed with empty pBI121 vector). To determine the expression of *PtrGT10* in DR and control plants, qRT-PCR was performed. At 48 h after transformation, the plants were grown under normal condition or treated with 200 mM mannitol for 24 h and 48 h, and the expression of *PtrGT10* in whole plants of DR and control plants were investigated. The expression levels of *PtrGT10* in DR plants were normalized by using that in control plants at time point of 24 h. Compared with in control plants, the expression of *PtrGT10* was significantly decreased in DR plants under both normal and osmotic stress conditions (Supplementary Fig. S5). These results indicated that the transient transformation system is suitable for function studies of *PtrGT10*.

### Biochemical staining and physiological measurement of *35S::PtrGT10SRDX* transformed plants

**Figure 9:**
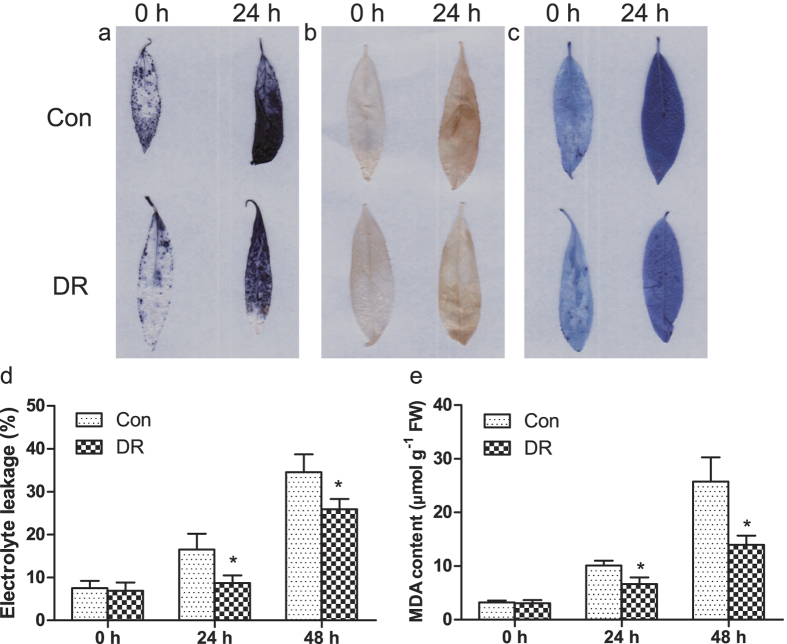
Histochemical staining and related physiological changes analyses of transformed *P. ussuriensis* plants. (**a,b**) The plants were stained with NBT (**a**) and DAB (**b**) to reveal the accumulation of O^2−^ and H_2_O_2_, respectively. (**c**) Analysis of cell death by Evans Blue staining. (**d**) Analysis of electrolyte leakage rate. (**e**) Analysis of MDA level. The asterisks indicate statistically significant differences between the relative expression of control and DR plants (*P < 0.05).

To study reactive oxygen species (ROS) accumulation, nitroblue tetrazolium (NBT) and 3,3-diamin-obenzidine (DAB) *in situ* staining on *Populus ussuriensis* were performed, which can stain two main ROS species, H_2_O_2_ and O^2−^. Both NBT and DAB staining showed that the DR plants (plants transform with *35S::PtrGT10SRDX* for dominant repressing *PtrGT10*) had lower H_2_O_2_ and O^2−^ levels compared with control plants under osmotic stress (Fig. 9a,b). These results suggested that the repression of *PtrGT10* could improve the ROS scavenging ability. Evans blue *in situ* staining on *P. ussuriensis* showed that cell death decreased in the DR plants compared with control plants under osmotic stress (Fig. 9c). An electrolyte leakage assay further confirmed these results. It showed that the electrolyte leakage rate was lower in DR plants than in control plants under osmotic stress (Fig. 9d). Under normal conditions, there was no difference in MDA levels between control and DR plants. However, under osmotic stress, the DR plants displayed the lower MDA content (Fig. 9e). These results indicated that the repression of *PtrGT10* could reduce cell death and MDA accumulation under osmotic stress.

## Discussion

Trihelix genes play a key role in plant physical development and in responses to environmental stimuli. In this study, we identified 56 trihelix genes in *P. trichocarpa*, each of which had at least one trihelix domain. The number of identified trihelix genes is much higher than that found in *Arabidopsis* and rice (30 and 31, respectively), reflecting differences between herbaceous and woody plants. Gene duplications are known to play a crucial role in genome expansions and realignments, which include tandem and segmental duplications^23^. According to our phylogenetic analysis, 15 paralogous pairs were identified, of which only one pair (*PtrGT53*/*54*) has undergone tandem duplication; the other 14 paralogous pairs were all segmental duplications, suggesting a high rate of segmental repetition, which is helpful for gene evolution.

Gene structural variation plays an important role in gene evolution, as integrations and realignments of gene fragments can cause exon/intron increases or decreases^24^. In this study, we identified such variation in *PtrGT42* and *PtrGT47* of the SIP1 subfamily, which each contained 17 exons, whereas most of the other genes in this subfamily contained only two or three exons. *PtrGT30, 35* and *40* also each contain six or seven exons. These results indicate that these specific genes have undergone a series of gene evolution events leading to exon increases and thus may be functionally different. Structures and conserved motifs were similar across most genes within a given subfamily, indicative of similar functions and stable evolution. However, there were also some special genes whose motifs differed from others in the same subgroup, such as *PtrGT33, 44* and *52*, which lacked motifs 6, 7 and 11. This result suggests that these genes have divergent functions compared with the other genes.

Promoter *cis*-elements are known to play important roles in response to biotic and abiotic stresses^25^. In this report, many biotic and abiotic stress-related and phytohormone-related *cis*-elements were identified in the promoters of trihelix family genes^26^, including ABRE, W-Box, DRE, ERE, G-Box and EIRE elements. *PtrGT14* and *PtrGT26* had 11 *cis*-elements, suggesting important functions under different stresses. In particular, *PtrGT14* was the only gene with a dehydration-responsive DRE *cis*-element. However, no obvious expression changes under osmotic stress were detected by qRT-PCR. This may be attributable to later gene upregulation after 24 h. *PtrGT4* had two SA- and stress-related *cis*-elements (TCA-element and TC-rich repeats). The qRT-PCR results showed that *PtrGT4* was induced not only by SA but also by ABA and MeJA, suggesting a close relationship between ABA and MeJA with SA. ELI-box3 and EIRE are fungal stress-related *cis*-elements that were present in six trihelix genes (*PtrGT13, 14, 15, 27, 32* and *34*). Our qRT-PCR results showed that three of these genes (*PtrGT13, 14, 32*) were upregulated during pathogen infection, while the other three genes showed no obvious change. This result indicates that some trihelix genes are involved in a different pathogen expression network.

From our study of exImage data, we found trihelix genes expressed in different tissues of *Populus*; however, there are obvious differences. *PtrGT9, 32, 45* and *47* were highly expressed in young leaves, though the expression of all but *PtrGT32* was less remarkable in mature leaves. Current information suggests that trihelix transcription factors regulate light-response genes^1^,^27^ and that loss of *AtGTL1* gene can negatively regulate water use efficiency by modulating stomatal density, thus leading to increased plant tolerance to water deficits^12^. These previous studies suggested relationships between trihelix genes and leaf stoma or light responses. Therefore, *PtrGT32*, which is highly expressed in both young leaves and mature leaves, may be related to stoma or photosynthesis through cooperation with other photosynthesis-related genes. In addition under drought conditions, roots can sense soil changes and send a series of signals to the shoots and leaves to reduce root damage^28^. In our study, *PtrGT4, 20, 31, 41* and *52* were highly expressed in roots, suggesting that these genes might enhance the ability of tissues to absorb water to adapt to the drought environment. The distinct expression patterns of the trihelix family members suggest a diversity of functions during plant growth and development.

Plants are frequently threatened by abiotic and biotic stresses that influence stress resistance or even cause fatal damage in trees^29^. However, many genes help plants adapt to these stresses via gene expression changes. Under osmotic conditions, 42 trihelix genes were induced, eight genes were downregulated, and six genes showed no obvious change. This result was broadly consistent with the exPlot data, which indicated that trihelix genes act synergistically and have important functions in drought stress. A soybean homolog (*Glyma10g34520*) of one of the upregulated genes (*PtrGT40*) was similarly upregulated under drought conditions. This finding suggests that the homologous genes have similar functions in different species. *PtrGT10* was suppressed by osmotic, consistent with a previous study^15^ that showed that poplar GT1 (*PtrGT10* in our study) functioned in drought tolerance. ABA is an important regulatory factor during drought stress^30^. SA and MeJA are well-known naturally occurring signaling molecules that play key roles in biotic stresses^24^,^31^. In our study, most of the trihelix genes were induced under ABA, SA and MeJA treatment, indicating that phytohormones regulate expression of trihelix genes in *P. trichocarpa*. However, different genes were induced by different phytohormones. For example, *PtrGT3* and *PtrGT13* were induced by ABA and MeJA, but suppressed by SA. *PtrGT9* was induced by ABA, but suppressed by SA and MeJA. These variations indicate that a broad set of hormonal signals exist in the trihelix gene family. *OsGTγ-1* (LOC_Os02g33770) was specifically upregulated by an ABA and SA stimulus^8^. However, the homologous gene in *P. trichocarpa (PtrGT13*) was only induced by ABA, indicating different expression patterns between woody plants and crops. Notably, *GmGT2A* and *GmGT2B*, which are orthologs of *PtrGT9* and *PtrGT24*, were induced by drought and ABA in soybean, suggesting that these two genes have similar responses to drought and ABA treatment in different species^13^. In our study, expression of most trihelix genes was induced by mannitol and phytohormone treatments within a short term (24 h), but only a few genes were induced long term (7 d). Among these long-time response genes, *PtrGT3, Ptrgt37* and *PtrGT42* were simultaneously upregulated under mannitol, ABA, SA and MeJA treatment, respectively. This result further suggests that these three genes are involved in biotic and abiotic stresses as well as long-term response genes to biotic and abiotic stresses.

*Alternaria alternate* causes poplar leaf blight, one of the most common diseases in Northeast China, and can cause serious economic impacts^32^. As little research has investigated the expression levels of trihelix genes during fungal infection of *Populus*, we carried out such an analysis based on the presence of fungi-related *cis*-elements in trihelix gene promoters and a related study in soybean^10^. Most of the genes with fungus-related *cis*-elements in their promoters were upregulated by *A. alternate* infection, except for *PtrGT15, PtrGT35* and *PtrGT44*, which were downregulated. Conversely, 24 genes that were upregulated by SA and MeJA stresses were also upregulated by *A. alternate* infection. This may indicate a relationship among SA, MeJA and fungi, where SA and MeJA have a regulatory function under this type of biotic stress (fungal infection), consistent with previous studies^24^,^31^. Based on expression profile analysis and our qRT-PCR study, 22 genes responded to all five stresses, 19 genes responded to four stresses, eight genes responded to three stresses, six genes responded to two stresses, and only one gene responded to one stress. These results indicate that a given trihelix gene may not be associated with a single type of stress. Follow-up studies should establish interactions among the particular pathways activated by biotic stress, abiotic stress and phytohormones.

Transient transformation system provides a powerful and convenient tool to investigate gene functions *in vivo,* avoiding difficult drawbacks that typically affect the stable transformation protocols, such as transformation efficiency, transformants selection, and regeneration^33^. A prior research has already demonstrated that the transient transformation system used in this study is reliable for plants stress tolerance studies^34^. In our study, the decreased expression levels in transient transformed plants (DR plants) also confirmed the reliability of gene function analysis. A previous study showed that overexpression of *PtrGT10* suppressed water use efficiency and drought tolerance by increasing stomatal density^15^. In this study, repression of *PtrGT10* in *P. ussuriensis* was related to reduce ROS accumulation, indicating that repression of *PtrGT10* is involved in ROS scavenging. The lower electrolyte leakage rate of DR plants indicated the decreased cell death under osmotic stress. Meanwhile, repression of *PtrGT10* was related to greatly decreased MDA content under mannitol condition, suggesting that lipid peroxidation in cell membrance was also decreased. Increased ROS scavenging is a common mechanism to induce stress tolerance in plants. Therefore, these results suggested that repression of *PtrGT10* could decrease cell death by increasing ROS scavenging ability and decreasing cell damage, thus increasing the osmotic stress tolerance.

MiRNAs have important functions in plant growth, development and abiotic stress responses. MiR172 is a key miRNA in flower development, regulating calyx and petal formation and developmental timing^35^. The targets of miR172 were identified in a previous study as AP2-like transcription factor genes^36^. Previous studies reported that AP2 regulates flower and seed development^37^. In recent years, some studies have shown that *miR172* is also involved in abiotic stress responses^38^. For example, it was significantly induced by salt in wheat^39^, but decreased under drought stress in rice^40^. Some studies have identified targets of miR172 during abiotic stress responses. Overexpression of soybean *gma-miR172c* in *Arabidopsis* resulted in reduced leaf water loss and an increased survival rate. Moreover, the target of *gma-miR172c, Glyma01g39520*, an AP2-like gene, can reduce tolerance to drought stress in *Arabidopsis* mutants^38^. *MiR172* downregulates *AP2* through transcript cleavage and translational repression in *A. thaliana*^41^. However, to date, no studies have reported interactions between miR172d and abiotic stress-related targets except for *AP2* genes. Our study provides a new target gene, *PtrGT10*, which is an osmotic-related gene in *P. trichocarpa*. In this study, *PtrGT10* may negatively regulate the osmotic stress response. Therefore, miR172d may regulate this new target gene, *PtrGT10*, as part of the osmotic response. However, whether the regulatory mechanism of miR172d involves translational repression of *PtrGT10* needs further study.

In this study, 56 trihelix genes were identified in the *Populus* genome and classified into five groups. The exon/intron structures and motif compositions of most trihelix genes were conserved in each subfamily, indicating their likely functional conservation. The genes were non-randomly distributed on 19 LGs and were predicted to be expressed in different tissues (mostly in leaves and roots) and under different stresses. Some genes were significantly induced under biotic or abiotic stresses and phytohormone treatments, which will be helpful for future functional studies aiming to reveal their divergent roles. Additionally, a new target of miR172d, *PtrGT10*, was identified, with the expression levels of *miR172d* and *PtrGT10* showing inverse trends under osmotic stress. Repression of *PtrGT10* could increase ROS scavenging ability and decrease cell death. These important discoveries may lay foundations for follow-up studies.

## Materials and Methods

### Identification of the trihelix gene family in *Populus*

The *P. trichocarpa* genome database Phytozome V10.3 and the NCBI database were searched to compile a cDNA library and a protein database. All of the obtained sequences were examined for the presence of trihelix domains (PF13837, SM00717) using Pfam 28.0^19^ and the SMART database^42^. The identified genes were compared in PlnTFDB V3.0^43^. Related trihelix genes in *Arabidopsis* and rice were downloaded from the *Arabidopsis* Information Resource (TAIR) and the Rice Information Resource, respectively. WOLF PSORT was used to predict subcellular localizations^44^. Physicochemical parameters were calculated using ExPASy.

### Phylogenetic analysis

Further multiple sequence alignments were performed by ClustalX 1.83^45^ and manually corrected using BioEdit 7.1^46^. Phylogenetic trees were constructed using MEGA 5.0 with the neighbor-joining (NJ) method, and the bootstrap test was conducted using 1,000 replicates^7^. Gene clusters of the homologs in the three species (*P. trichocarpa, A. thaliana* and *O. sativa*) were identified based on the NCBI database (http://www.ncbi.nlm.nih.gov/).

### Gene structure and conserved motif analysis

The trihelix gene exon/intron organization was determined using the GSDS 2.0 program^47^. The MEME system (v4.10.2)^48^ was used to identify conserved motifs. MEME was run locally with the following parameters: number of repetitions, any; maximum number of motifs, 15; and the optimum motif widths, between 6 and 200 residues. In addition, structural motif annotation was performed with Pfam and the SMART database.

### Chromosomal location analysis

The chromosomal locations were identified and plotted with the PopGenIE v3.0 database^49^. Tandem gene duplications in *Populus* were identified based on the same criteria described in rice (http://compbio.dfci.harvard.edu/tgi/). Genes separated by ≤5 gene loci in a range of 100 kb physical distance were considered tandem duplicates^50^. The synonymous (Ks) and nonsynonymous substitution (Ka) rates were calculated based on a previous study^51^.

### Promoter *cis*-element identification

Promoter sequences located 2 kb upstream of the translation start site were obtained from the Phytozome v10.3 database and analyzed with PlantCARE^52^.

### Gene Ontology (GO) annotation

Functional annotation of trihelix sequences and the associated analyses were performed using Blast2GO v3.0^53^. The protein sequences were imported into the Blast2GO program to associate GO terms. The GO categories were placed in a hierarchical classification with three independent classes, namely, biological processes, cellular components and molecular functions.

### exImage and exPlot analysis

The exImage tool was used to analyze expression levels across different tissues of *P. trichocarpa* using the NCBI GEO number GSE6422^20^ as a control. The exPlot tool in PopGenIE v3.0 was used to visualize the explot of genes in different tissues. The data can be directly obtained using the accession numbers.

### Plant materials and stress treatments

*In-vitro* plantlets of *P. trichocarpa* (genotype Nisqually-1) were cultured in half-strength Murashige and Skoog (1/2 MS) medium^54^ under long-day conditions (16 h light/8 h dark) at a temperature of 25 °C. Plants were grown in 250-ml erlenmeyer flasks containing 100 ml medium and were subcultured at 4-week intervals. In a preliminary experiment, we evaluated the effect of different concentrations of mannitol on growth of *in vitro* plants. The results showed that 150 mM mannitol could reduce but not abolish growth of *in vitro* plants. Thus, 150 mM mannitol was use as moderate osmotic stress treatment in this study. For phytohormone treatments, the plants were cultured in 1/2 MS medium with 200 μM ABA^55^, 100 μM SA^56^ or 100 μM MeJA^57^, according to a previous study. Prior to this, we also confirmed the ABA, SA and MeJA concentrations mentioned above, which can reduce growth but not induce wilting or necrosis within 7 d. All media were adjusted to pH 5.8 before addition of 0.6% plant agar (Biotopped, Beijing, China), and then sterilized by autoclaving at 121 °C for 15 min. Mannitol was added to medium before autoclaved sterilization. The ABA, SA and MeJA stock solutions were filter sterilized and added to the medium after autoclaving, respectively. To induce fungal infection, mycelial plugs of *Alternaria alternate* (the pathogen of *Populus* leaf blight) were placed on excised leaves, which were then cultured in medium, according to a previous study^32^. The 3, 6, 12 and 24 h time points were chosen to obtain early responsive genes, and the 7 d point for late responsive genes. Fungal infections lasted for 0 h, 3 h, 6 h, 12 h and 24 h. Three different plants were collected from each treatment time point. Three independent biological replicates were performed for each treatment. All samples were subsequently frozen and stored at −80 °C for RNA isolation.

### RNA isolation and qRT-PCR analysis

Total RNA was extracted as previously described^58^. cDNA was synthesized based on the manufacturer’s instructions for the ReverTra Ace qPCR RT Master Mix with a gDNA Remover Kit (TOYOBO, Osaka, Japan). Primer Premier 5 was used to design gene-specific primers. Each primer was checked by BLAST for specificity, and a melting curve analysis was performed. The *actin* gene (XM_002298674) of *P. trichocarpa* was used as the reference gene^59^. To verify that the treatments were working well, the homologous genes in poplars of *RD29A* (POPTR_0012s13180), *RAB18* (POPTR_0002s07510), *PR1* (POPTR_0009s08670) and *PR4* (POPTR_0013s03890) were used as marker genes for mannitol, ABA, SA and MeJA treatments, respectively. Primer details are in Supplementary Table S7. qRT-PCR was performed in a volume of 20 μL with 10 μL of 2 × SYBR Premix, 6 μL of ddH_2_O, 2 μL of template and 1 μL of each specific primer (final concentration 10 μM). The PCR conditions and relative expression levels were calculated as previously described^60^. Three biological replicates were used for each sample.

### Cleavage validation of miRNA targets and qRT-PCR analysis

The psRNATarget online tool^61^ and previous results^62^ were used to search for the presence of microRNA targets. The modified RNA ligase-mediated rapid amplification of 5′ cDNAs method (5′RLM-RACE) was performed using the GeneRacer kit (Invitrogen, Carlsbad, CA, USA). Total RNA was isolated from *P. trichocarpa* leaves under osmotic stress. A poly(A) tail was added to the 3′ end prior to transcription. A SYBR Green miRNA qPCR Detection Kit (Bioteck, Beijing, China) was used to detect miRNA expression levels, with 5.8S rRNA used as the endogenous reference. All reactions were run in triplicate. All primers mentioned above are listed in Supplementary Table S7.

### Cloning of the *PtrGT10* gene and plant transformation

To verify the function of trihelix genes, we constructed repression vectors of *PtrGT10* using the method described in previous study^63^. The full-length *PtrGT10* was amplified with gene-specific primers (see in Supplementary Table S7) by RT-PCR with 2 μL cDNA from *P. trichocarpa* leaves. The PCR reaction was performed with LA Taq DNA polymerase (TaKaRa, Dalian, China) in a total volume of 50 μL with an initial denaturing step at 94 °C for 3 min, 35 cycles of 94 °C for 30s, 60 °C for 30s and 72 °C for 3 min, and a final extension step at 72 °C for 7 min. The *PtrGT10* dominant repression constructs were created by fusing the full-length *PtrGT10* cDNA in frame with the SRDX repression sequence, which was ligated downstream of the 35S promoter in pBI121^63^. *Agrobacterium*-mediated transient transformation of whole *P. ussuriensis* plantlets, as well as the osmotic treatment, were performed using a previous method^34^. The 4-week-old WT plantlets with similar size were used for transformation. Two kinds of transiently transfected plantlets were generated, i.e., plants transform with *35S::PtrGT10SRDX* for dominant repressing *PtrGT10* (DR) and control (transformed with empty pBI121 vector).

### Biochemical staining and physiological measurement of *35S::PtrGT10SRDX* transformed plants

After culture on agar medium, the plantlets were harvested for biochemical staining. *P. ussuriensis* repressing plants and control plants subjected to the 200 mM mannitol were infiltrated with DAB solution NBT solution following the procedure described by ref. 64. Cell death was examined by Evans blue staining as described by ref. 65. In each biochemical staining experiment, at least ten plantlets were used.

After being cultured on agar medium, the plants were moved to new medium with 200 mM mannitol for stress induction for 24 h and 48 h. Then, the plants were harvested for physiological analyses. MDA measurement was conducted as in ref. 66, while the electrolyte leakage measurement was performed as described by ref. 34. Biological replicates were performed in triplicate.

## Additional Information

**How to cite this article**: Wang, Z. *et al*. Comprehensive analysis of trihelix genes and their expression under biotic and abiotic stresses in *Populus trichocarpa. Sci. Rep.* **6**, 36274; doi: 10.1038/srep36274 (2016).

**Publisher’s note**: Springer Nature remains neutral with regard to jurisdictional claims in published maps and institutional affiliations.

## Supplementary Material

Supplementary Information

## Figures and Tables

**Table 1:** Divergence between trihelix gene pairs.

Paralogous pairs	Ks	Ka	Ka/Ks	Duplicate type
*PtrGT1-PtrGT23*	0.2596	0.0912	0.3512	Segmental
*PtrGT2-PtrGT22*	0.5016	0.1202	0.2395	Segmental
*PtrGT3-PtrGT21*	0.3401	0.0834	0.2454	Segmental
*PtrGT4-PtrGT20*	0.2943	0.0496	0.1684	Segmental
*PtrGT5-PtrGT19*	0.3053	0.0447	0.1463	Segmental
*PtrGT6-PtrGT18*	0.2970	0.0524	0.1763	Segmental
*PtrGT8-PtrGT16*	0.3541	0.0777	0.2195	Segmental
*PtrGT11-PtrGT27*	0.1915	0.0935	0.4882	Segmental
*PtrGT12-PtrGT26*	0.3171	0.0863	0.2721	Segmental
*PtrGT13-PtrGT25*	0.2668	0.0475	0.1782	Segmental
*PtrGT14-PtrGT46*	0.2906	0.0492	0.1695	Segmental
*PtrGT31-PtrGT50*	0.2912	0.0671	0.2303	Segmental
*PtrGT36-PtrGT39*	0.3374	0.1374	0.4073	Segmental
*PtrGT37-PtrGT38*	0.2574	0.0434	0.1685	Segmental
*PtrGT53-PtrGT54*	0.1104	0.0776	0.7029	tandem

Gene pairs were identified at the terminal nodes (>90% identical) of the phylogenetic tree. Synonymous (Ks) and nonsynonymous substitution (Ka) rates are presented for each pair. Gene pairs created by tandem duplication or segmental duplication are indicated in the table.
