# Supplementary material for: Comprehensive analysis of trihelix genes and their expression under biotic and abiotic stresses in Populus trichocarpa
Source: Sci Rep. 2016 Oct 26;6:36274. doi: 10.1038/srep36274 (PMC5080644; doi:10.1038/srep36274)
Supplement: Supplementary Information [file srep36274-s1.pdf]

Comprehensive analysis of trihelix genes and their expression under biotic and abiotic stresses in *Populus trichocarpa*

Zhanchao Wang[1&], Quangang Liu[1&], Hanzeng Wang[1], Haizhen Zhang[1], Xuemei Xu[2], Chenghao Li[1*], Chuanping Yang[1*]

[1]State Key Laboratory of Tree Genetics and Breeding, Northeast Forestry University, 26 Hexing Road, Harbin 150040, China (e-mail: jiajia900325@126.com; liuquangang007@126.com; 15526935551@163.com; snowsun04006@hotmail.com);

[2]Library of Northeast Forestry University, 26 Hexing Road, Harbin 150040, China (e-mail: chli0@daum.net)

[*]Corresponding author (e-mail: chli0@163.com; yangchuanpingnefu@163.com).

[&]These authors contributed equally to this work as co-first authors.

Supplementary Figure S1. Multiple sequence alignment of trihelix genes in *Populus*. Conserved amino acid residues are marked with specific letters.

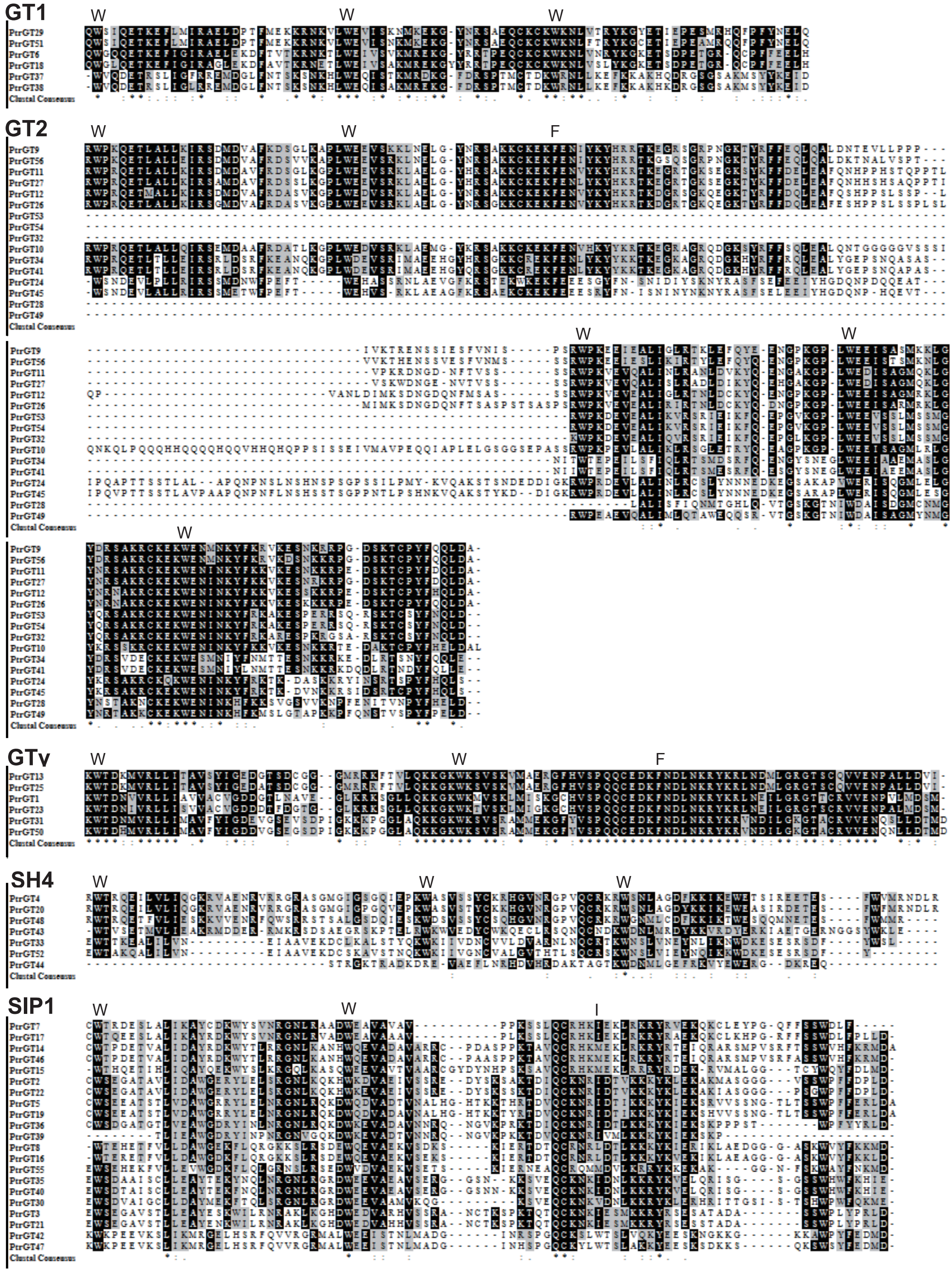

Supplementary Figure S2. Explot analysis of *Populus* trihelix genes in leaves and roots under drought stress. Yellow and black indicate high and low levels of transcript abundances, respectively.

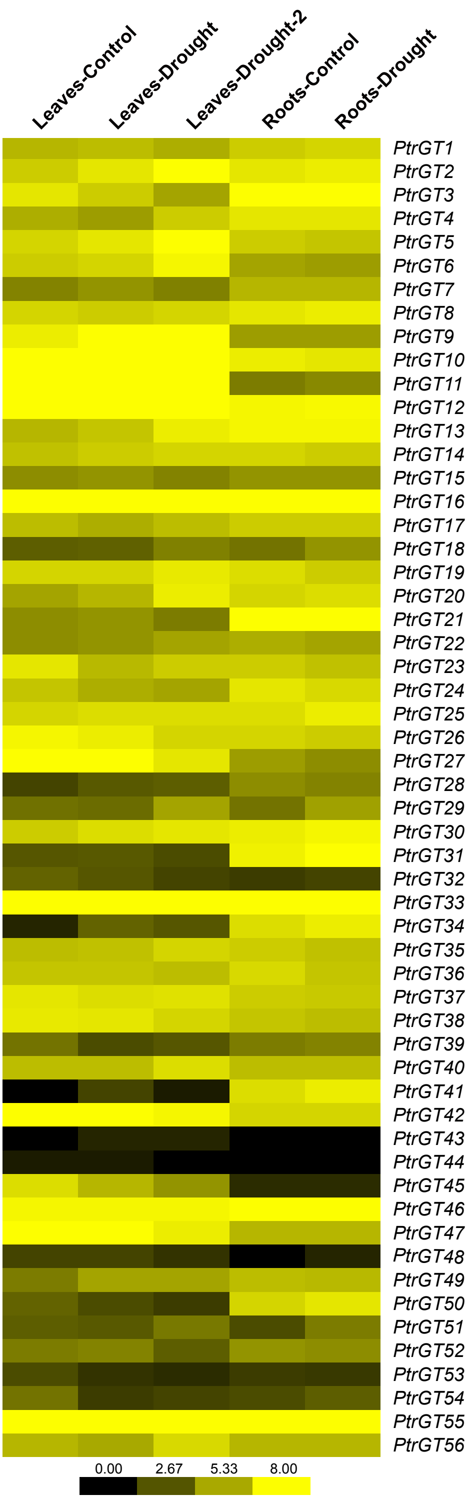

Supplementary Figure S3. The expression levels of marker genes during mannitol, ABA, SA and MeJA treatments.

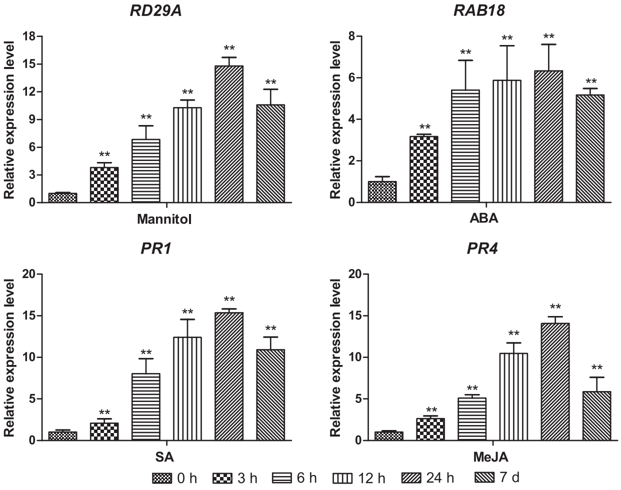

Supplementary Figure S4. The original sequencing peaks of 5' RLM-RACE.

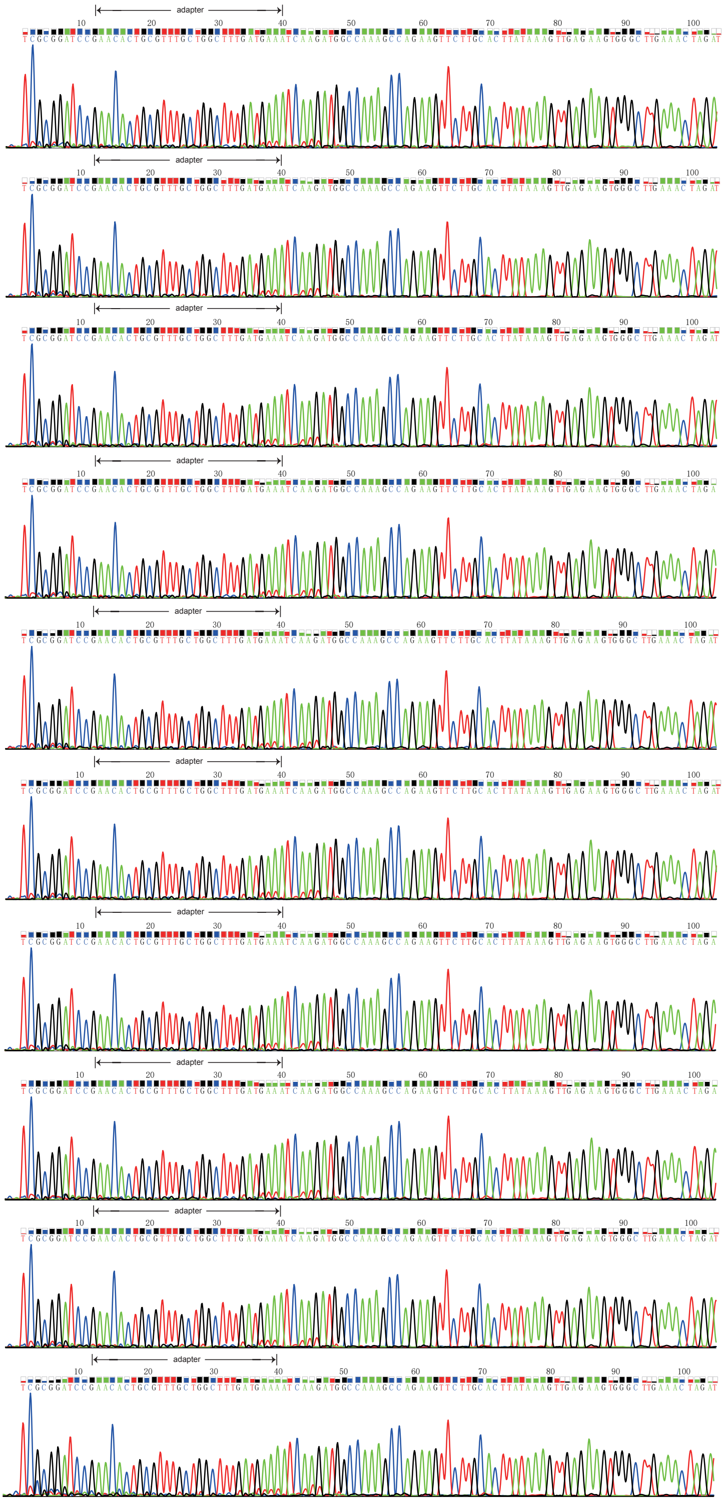

Supplementary Figure S5. The expression levels of *PtrGT10* in control and DR plants under normal and osmotic conditions.

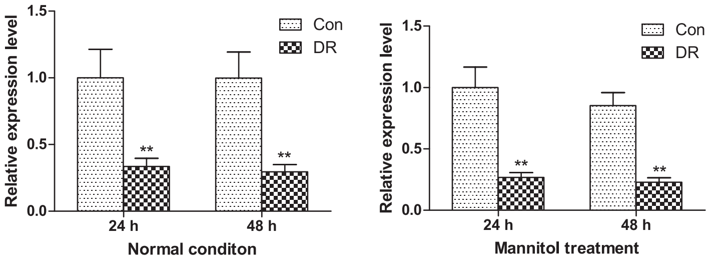

Supplementary Table S1. Alternative splicing of *Populus* trihelix genes.

Gene name	Primary transcript	Other transcripts
PtrGT9	Potri.001G309100.1	Potri.001G309100.2
PtrGT10	Potri.001G454500.1	Potri.001G454500.2
PtrGT20	Potri.003G163000.1	Potri.003G163000.2
PtrGT21	Potri.003G195300.1	Potri.003G195300.2
PtrGT24	Potri.005G051700.1	Potri.005G051700.2, Potri.005G051700.3
PtrGT25	Potri.005G191700.1	Potri.005G191700.2
PtrGT28	Potri.006G005300.1	Potri.006G005300.2
PtrGT30	Potri.006G117300.1	Potri.006G117300.2
PtrGT32	Potri.006G221400.1	Potri.006G221400.2
PtrGT33	Potri.006G279100.1	Potri.006G279100.2
PtrGT35	Potri.008G027000.1	Potri.008G027000.2
PtrGT36	Potri.008G071300.1	Potri.008G071300.2
PtrGT37	Potri.008G179700.1	Potri.008G179700.2, Potri.008G179700.3
PtrGT40	Potri.010G233500.1	Potri.010G233500.2
PtrGT45	Potri.013G039100.1	Potri.013G039100.2
PtrGT47	Potri.015G093600.1	Potri.015G093600.2
PtrGT55	Potri.018G075500.1	Potri.018G075500.2

Supplementary Table S2. List of trihelix genes identified in *Populus* and their sequence characteristics.

Gene name	Accession number	TF ID	NCBI locus ID	*Arabidopsis* ortholog locus	Length (a.a.)	M.W. (Da)	pI	Localization
PtrGT1	POPTR_0001s05670	Potri.001G019200.1	XP_002299233.1	AT1G21200.1	459	51475.2	5.53	nucleus
PtrGT2	POPTR_0001s06330	Potri.001G026000.1	XP_002297748.1	AT3G14180.1	325	36419.90	4.94	nucleus
PtrGT3	POPTR_0001s06580	Potri.001G028400.1	XP_002299199.1	AT3G54390.1	352	38717.00	10.36	nucleus
PtrGT4	POPTR_0001s13060	Potri.001G066900.1	XP_002299351.1	AT2G33550.1	346	38046.60	6.20	nucleus
PtrGT5	POPTR_0001s08350	Potri.001G113600.1	XP_002299577.1	AT3G58630.1	302	34336.30	5.25	nucleus
PtrGT6	POPTR_0001s01210	Potri.001G129900.1	XP_002326589.1	AT2G38250.1	296	35117.60	8.47	nucleus
PtrGT7	POPTR_0001s15070	Potri.001G150600.1	XP_002326679.1	AT2G44730.1	471	52310.00	8.29	cytoplasm
PtrGT8	POPTR_0001s18000	Potri.001G179800.1	XP_002298141.1	AT3G24490.1	334	38374.20	4.70	nucleus
PtrGT9	POPTR_0001s31660	Potri.001G309100.1	XP_002298711.1	AT1G76880.1	502	57379.40	5.34	nucleus
PtrGT10	POPTR_0001s45870	Potri.001G454500.1	XP_002300534.1	AT1G33240.1	794	86862.50	5.42	nucleus
PtrGT11	POPTR_0002s06900	Potri.002G068400.1	XP_002300920.1	AT1G76890.2	593	66528.00	5.39	nucleus
PtrGT12	POPTR_0002s06920	Potri.002G068600.1	XP_002302180.1	AT1G76880.1	605	67207.10	7.94	nucleus
PtrGT13	POPTR_0002s06930	Potri.002G068700.1	XP_002302181.1	AT1G21200.1	420	48443.90	6.51	nucleus
PtrGT14	POPTR_0002s14030	Potri.002G139500.1	XP_002301240.1	AT2G44730.1	365	40637.90	9.96	nucleus
PtrGT15	POPTR_0002s24480	Potri.002G243300.1	XP_002301790.1	AT3G24860.1	294	33991.10	5.93	nucleus
PtrGT16	POPTR_0003s05470	Potri.003G056000.1	XP_002303280.1	AT3G24490.1	413	47342.30	4.29	nucleus
PtrGT17	POPTR_0003s08210	Potri.003G083800.1	XP_002329555.1	AT2G44730.1	457	50734.40	8.30	nucleus
PtrGT18	POPTR_0003s10360	Potri.003G104500.1	XP_002304391.1	AT2G38250.1	300	35284.80	8.06	nucleus
PtrGT19	POPTR_0003s11850	Potri.003G118600.1	XP_002303549.1	AT3G58630.1	290	32978.00	5.80	nucleus
PtrGT20	POPTR_0003s16150	Potri.003G163000.1	XP_002299351.1	AT2G33550.1	342	37593.10	7.15	nucleus
PtrGT21	POPTR_0003s19470	Potri.003G195300.1	XP_002303935.1	AT3G54390.1	352	38706.90	10.23	nucleus
PtrGT22	POPTR_0003s19840	Potri.003G198800.1	XP_002304787.1	AT3G14180.1	365	40652.20	9.19	nucleus
PtrGT23	POPTR_0003s20370	Potri.003G204700.1	XP_002303892.1	AT1G21200.1	454	51187.00	6.56	nucleus
PtrGT24	POPTR_0005s05330	Potri.005G051700.1	XP_002319635.1	AT5G28300.1	626	71086.90	6.64	nucleus
PtrGT25	POPTR_0005s21390	Potri.005G191700.1	XP_002306694.1	AT1G21200.1	438	50474.00	6.85	nucleus
PtrGT26	POPTR_0005s21410	Potri.005G191900.1	XP_002306695.1	AT1G76880.1	608	67986.00	6.54	nucleus
PtrGT27	POPTR_0005s21420	Potri.005G192000.1	XP_002307497.1	AT1G76890.2	587	65833.60	6.35	nucleus
PtrGT28	POPTR_0006s00710	Potri.006G005300.1	XP_002308761.1	AT1G76890.2	295	32572.60	7.02	nucleus
PtrGT29	POPTR_0006s10240	Potri.006G101400.1	XP_002308224.1	AT5G01380.1	274	32831.00	8.12	nucleus
PtrGT30	POPTR_0006s11850	Potri.006G117300.1	XP_006381330.1	AT3G10030.1	492	53875.50	6.63	cytoplasm
PtrGT31	POPTR_0006s11870	Potri.006G117500.1	XP_002326236.1	AT3G10040.1	483	55719.30	5.40	nucleus
PtrGT32	POPTR_0006s23800	Potri.006G221400.1	XP_002308526.1	AT1G76890.2	618	70311.00	6.31	nucleus
PtrGT33	POPTR_0006s29340	Potri.006G279100.1	XP_002330777.1	AT4G31270.1	459	53622.60	4.99	nucleus
PtrGT34	POPTR_0008s02580	Potri.008G025600.1	XP_002311966.1	AT5G03680.1	571	66028.00	6.60	nucleus
PtrGT35	POPTR_0008s02730	Potri.008G027000.1	XP_002311975.1	AT3G10030.1	499	54134.90	8.09	nucleus
PtrGT36	POPTR_0008s07140	Potri.008G071300.1	XP_002311242.1	AT5G05550.2	251	28839.60	9.49	nucleus
PtrGT37	POPTR_0008s17980	Potri.008G179700.1	XP_002312646.1	AT1G13450.1	385	44093.40	6.66	nucleus
PtrGT38	POPTR_0010s06510	Potri.010G055000.1	XP_002315635.1	AT1G13450.1	384	44078.20	6.52	nucleus
PtrGT39	POPTR_0010s19350	Potri.010G186200.1	XP_002316198.1	AT5G05550.2	279	33004.80	10.19	nucleus

PtrGT40	POPTR_0010s23970	Potri.010G233500.1	XP_002315355.1	AT3G10030.1	472	50892.90	8.31	nucleus
PtrGT41	POPTR_0010s24140	Potri.010G235000.1	XP_002316512.1	AT5G03680.1	575	66590.50	6.19	nucleus
PtrGT42	POPTR_0012s09780	Potri.012G095800.1	XP_002318122.1	AT5G63420.1	916	101174.00	8.59	chloroplast
PtrGT43	POPTR_0012s11820	Potri.012G117500.1	XP_002332145.1	AT2G35640.1	355	39719.70	8.36	nucleus
PtrGT44	POPTR_0012s14050	Potri.012G132300.1	XP_002318853.1	AT2G33550.1	994	111294.00	7.17	nucleus
PtrGT45	POPTR_0013s03670	Potri.013G039100.1	XP_002319635.1	AT5G28300.1	647	73253.40	6.69	nucleus
PtrGT46	POPTR_0014s05010	Potri.014G051200.1	XP_002327024.1	AT2G44730.1	343	38131.60	10.63	chloroplast
PtrGT47	POPTR_0015s10570	Potri.015G093600.1	XP_002318122.1	AT5G63420.1	890	98789.00	8.34	chloroplast
PtrGT48	POPTR_0015s11710	Potri.015G105800.1	XP_002322303.1	AT2G33550.1	299	34823.90	7.38	nucleus
PtrGT49	POPTR_0016s00760	Potri.016G005900.1	XP_002323120.1	AT5G47660.1	363	39937.60	5.07	nucleus
PtrGT50	POPTR_0016s11010	Potri.016G096300.1	XP_002326236.1	AT3G10040.1	470	53990.00	6.39	nucleus
PtrGT51	POPTR_0016s12470	Potri.016G117300.1	XP_002322985.1	AT5G01380.1	279	33431.10	9.69	nucleus
PtrGT52	POPTR_0018s04010	Potri.018G002800.1	XP_002332103.1	AT4G31270.1	319	36550.30	5.11	nucleus
PtrGT53	POPTR_0310s00200	Potri.018G049500.1	XP_002324192.1	AT1G76890.2	316	36739.80	9.89	peroxisomal
PtrGT54	POPTR_0018s05520	Potri.018G049700.1	XP_002324190.1	AT1G76890.2	610	70003.40	6.45	nucleus
PtrGT55	POPTR_0018s06120	Potri.018G075500.1	XP_002324407.1	AT3G24490.1	354	41349.40	4.32	nucleus
PtrGT56	POPTR_0019s02650	Potri.019G010200.1	XP_002331882.1	AT1G76890.2	496	56887.80	5.02	nucleus

Supplementary Table S3. Motif sequences of trihelix genes.

Motif	Width (a.a.)	Best possible match	Domain
1	21	GYHRSAKQCKCKWENLNKYYK	the third α-helix
2	26	NCWPEQETLALIDIWSEMWIWFNRGN	the first α-helix
3	50	WKDFFERLMKKVMEKQEMMHNKFLETIEKCEHERMCREEAWRMQEMARIN	NA
4	41	KGPLWEEVSRKLAELGYNRSAKKCKEKFENVYKYHKRTKEG	the second α-helix in GT2
5	15	STWPYFHQLDALYNG	the fourth amphipathic α-helix
6	35	MLADAIKWFGEIYVRIESMKMQQMVELEKMRMEFQ	NA
7	15	KGPLWEEVSAAMSEK	NA
8	29	REHEIWAHERAWAAARDAAIMAFLQKITG	NA
9	29	SSSRWPKVEILALIQLRTNLECRYQENGP	NA
10	50	HSTTDKGKKGSPWQRMKWTDNMVRLLITAVFYIGDDGTSDCGGGMRRKFP	the first α-helix in GTγ
11	41	WEQKQWMKKRTLQLEEQQVNIQCQAFELEKQRFKWVRFCSK	NA
12	28	RLNDILGRGTSCRVVENPALLDTMDYLT	NA
13	50	FFQQQQQMEMQWREVFEAREHERQMFEQEWRQSMEKLENERLMMEQRWRE	coiled coil
14	29	RTGKQEGKTYRFFDQLEAFQNHPPHLSPP	the third α-helix in GT2
15	46	KEWEKEIRNETEFFWFMRNELRREFKLPGFFDREVYYILDGYMWTV	the extended third trihelix

Supplementary Table S4. Phytohormone- and abiotic stress-related *cis*-elements.

Element	Core sequence	Function annotation
ABRE	TACGGTC	Response to abscisic acid
CGTCA-motif	CGTCA	Response to MeJA
ERE	ATTTCAAA	Response to ethylene
G-Box	CACGTT	Response to MeJA
TCA-element	GAGAAGAATA	Response to salicylic acid
TGACG-motif	TGACG	Response to MeJA
W-Box	TTGACC	WRKY binding site involved in abiotic stress responsiveness
HSE	AAAAAATTTC	Response to heat stress
MBS	TAACTG	MYB binding site involved in drought-inducibility
LTR	CCGAAA	Response to low-temperature stress
DRE	TACCGACAT	Response to dehydration, low-temp, salt stresses
TC-rich repeats	ATTCTCTAAC	Involve in defense and stress responsiveness
EIRE	TTCGACC	Response to fungus
ELI-box3	AAACCAATT	Response to fungus

Supplementary Table S5. Numbers of *cis*-elements in trihelix genes.

Gene name	*Cis*-elements related to phytohormone						*Cis*-elements related to abiotic stress responsiveness						Cis-elements related to Fungi	
	ABRE	CGTCA-motif	ERE	G-Box	TCA-element	TGACG-motif	DRE	HSE	LTR	MBS	TC-rich repeats	W box	EIRE	ELI-box3
PtrGT1	●	●		●	●	●		●	●	●	●			
PtrGT2	●	●			●	●		●	●		●			
PtrGT3	●		●	●	●	●		●		●	●			
PtrGT4					●						●			
PtrGT5		●	●	●		●				●		●		
PtrGT6		●	●			●		●		●	●			
PtrGT7		●		●	●	●				●	●	●		
PtrGT8					●			●		●	●	●		
PtrGT9		●		●		●		●				●		
PtrGT10	●		●	●				●			●			
PtrGT11	●			●				●		●	●	●		
PtrGT12	●	●		●	●	●		●	●	●		●		
PtrGT13	●		●	●	●			●			●	●	●	
PtrGT14		●	●		●	●	●	●	●	●	●	●		●
PtrGT15		●				●		●	●		●	●	●	
PtrGT16		●		●		●		●			●			
PtrGT17		●	●	●	●	●		●	●	●	●	●		
PtrGT18	●			●	●	●		●	●		●			
PtrGT19	●	●		●	●	●				●	●			
PtrGT20				●				●		●	●	●		
PtrGT21	●	●		●		●		●		●	●	●		
PtrGT22	●			●	●			●	●	●	●			
PtrGT23	●	●		●	●	●		●	●	●	●			
PtrGT24	●	●	●	●	●	●		●	●	●				
PtrGT25	●	●	●	●	●	●		●	●		●			
PtrGT26	●	●	●	●	●	●		●	●	●	●	●		
PtrGT27	●	●	●	●	●	●					●		●	
PtrGT28			●	●	●			●	●		●			
PtrGT29	●		●	●	●	●		●	●	●	●	●		
PtrGT30	●			●				●	●			●		
PtrGT31	●		●	●	●			●		●	●			
PtrGT32		●	●	●		●		●	●	●	●	●		●
PtrGT33	●	●				●		●	●	●	●			
PtrGT34	●			●					●	●	●	●		
PtrGT35		●	●		●	●		●	●	●		●		●
PtrGT36	●	●		●	●	●		●	●	●	●	●		
PtrGT37	●			●		●		●		●	●	●		
PtrGT38		●	●	●	●	●		●		●	●	●		
PtrGT39		●				●		●	●	●		●		

PtrGT40	●	●	●	●		●			●	●	●	
PtrGT41		●	●	●		●	●		●		●	
PtrGT42	●	●	●	●	●	●	●		●	●	●	
PtrGT43		●		●	●	●	●	●	●	●	●	
PtrGT44		●	●		●	●	●			●		●
PtrGT45	●						●	●	●	●	●	
PtrGT46	●		●	●			●	●		●		
PtrGT47	●	●	●	●	●	●	●		●		●	
PtrGT48	●	●	●	●	●	●		●	●	●		
PtrGT49		●			●	●		●	●	●	●	
PtrGT50			●	●	●		●		●	●	●	
PtrGT51	●			●	●	●			●			
PtrGT52	●	●		●		●	●		●	●	●	
PtrGT53		●	●		●	●	●	●				
PtrGT54		●		●	●	●	●		●	●		
PtrGT55				●	●		●		●		●	
PtrGT56		●		●	●	●	●		●			

Supplementary Table S6. Details of the Gene Ontology annotation.

Seq. Name	Annotation number	GOs
PtrGT1	1	F:chromatin binding
PtrGT2	8	C:nucleus; F:DNA binding; F:sequence-specific DNA binding transcription factor activity; P:transcription, DNA-templated; P:embryo development ending in seed dormancy; P:seed maturation; P:post-translational protein modification; P:positive regulation of transcription, DNA-templated
PtrGT3	3	P:regulation of transcription, DNA-templated; F:sequence-specific DNA binding transcription factor activity; F:chromatin binding
PtrGT4	1	F:DNA binding
PtrGT5	2	P:regulation of transcription, DNA-templated; F:sequence-specific DNA binding transcription factor activity
PtrGT6	6	C:nucleus; F:chromatin binding; F:sequence-specific DNA binding transcription factor activity; F:sequence-specific DNA binding; P:transcription, DNA-templated; P:regulation of transcription, DNA-templated
PtrGT7	3	F:chromatin binding; C:vacuole; C:nucleus
PtrGT8	2	F:DNA binding; F:chromatin binding
PtrGT9	2	F:DNA binding; F:chromatin binding
PtrGT10	7	F:DNA binding; F:chromatin binding; P:cellular component organization; P:single-organism cellular process; P:negative regulation of nucleobase-containing compound metabolic process; P:regulation of multicellular organismal development; P:negative regulation of cellular macromolecule biosynthetic process
PtrGT11	2	F:DNA binding; F:chromatin binding
PtrGT12	2	F:DNA binding; F:chromatin binding
PtrGT13	1	F:chromatin binding
PtrGT14	3	F:chromatin binding; F:sequence-specific DNA binding transcription factor activity; P:regulation of transcription, DNA-templated
PtrGT15	2	F:sequence-specific DNA binding transcription factor activity; P:regulation of transcription, DNA-templated
PtrGT16	2	F:DNA binding; F:chromatin binding
PtrGT17	1	F:chromatin binding
PtrGT18	6	C:nucleus; F:chromatin binding; F:sequence-specific DNA binding transcription factor activity; F:sequence-specific DNA binding; P:transcription, DNA-templated; P:regulation of transcription, DNA-templated
PtrGT19	2	P:regulation of transcription, DNA-templated; F:sequence-specific DNA binding transcription factor activity
PtrGT20	1	F:DNA binding
PtrGT21	2	F:sequence-specific DNA binding transcription factor activity; P:regulation of transcription, DNA-templated
PtrGT22	0	-
PtrGT23	1	F:chromatin binding
PtrGT24	1	F:chromatin binding

PtrGT25	1	F:chromatin binding
PtrGT26	2	F:DNA binding; F:chromatin binding
PtrGT27	2	F:DNA binding; F:chromatin binding
PtrGT28	4	F:chromatin binding; F:DNA binding; P:regulation of transcription, DNA-templated; F:sequence-specific DNA binding transcription factor activity
PtrGT29	6	C:nucleus; F:chromatin binding; F:sequence-specific DNA binding transcription factor activity; F:sequence-specific DNA binding; P:transcription, DNA-templated; P:regulation of transcription, DNA-templated
PtrGT30	7	C:cytoplasm; F:chromatin binding; F:UMP kinase activity; P:nuclear-transcribed mRNA catabolic process; P:pyrimidine nucleotide biosynthetic process; P:nucleotide phosphorylation; P:photoperiodism, flowering
PtrGT31	4	P:response to stress; P:pattern specification process; P:regulation of cellular metabolic process; P:meristem development
PtrGT32	1	F:chromatin binding
PtrGT33	6	F:chromatin binding; P:regulation of transcription, DNA-templated; P:post-translational protein modification; C:plasmodesma; P:positive regulation of transcription, DNA-templated; F:sequence-specific DNA binding transcription factor activity
PtrGT34	1	F:chromatin binding
PtrGT35	5	C:cytoplasm; F:chromatin binding; F:UMP kinase activity; P:pyrimidine nucleotide biosynthetic process; P:nucleotide phosphorylation
PtrGT36	0	-
PtrGT37	6	C:nucleus; F:DNA binding; F:chromatin binding; F:sequence-specific DNA binding transcription factor activity; P:transcription, DNA-templated; P:regulation of transcription, DNA-templated
PtrGT38	6	C:nucleus; F:DNA binding; F:chromatin binding; F:sequence-specific DNA binding transcription factor activity; P:transcription, DNA-templated; P:regulation of transcription, DNA-templated
PtrGT39	0	-
PtrGT40	7	C:cytoplasm; F:chromatin binding; F:UMP kinase activity; P:nuclear-transcribed mRNA catabolic process; P:pyrimidine nucleotide biosynthetic process; P:nucleotide phosphorylation; P:photoperiodism, flowering
PtrGT41	1	F:chromatin binding
PtrGT42	7	C:chloroplast; F:DNA binding; F:chromatin binding; F:RNA binding; F:metal ion binding; P:photosynthesis; P:cellular respiration
PtrGT43	1	F:DNA binding
PtrGT44	3	F:kinase activity; P:phosphorylation; P:carpel morphogenesis
PtrGT45	1	F:chromatin binding
PtrGT46	1	F:chromatin binding
PtrGT47	7	C:chloroplast; F:DNA binding; F:chromatin binding; F:RNA binding; F:metal ion binding; P:photosynthesis; P:cellular respiration
PtrGT48	1	P:regulation of transcription, DNA-templated
PtrGT49	2	F:DNA binding; F:chromatin binding
PtrGT50	4	P:response to stress; P:pattern specification process; P:regulation of cellular metabolic process; P:meristem development
PtrGT51	6	C:nucleus; F:chromatin binding; F:sequence-specific DNA binding transcription factor activity; F:sequence-specific DNA binding; P:transcription, DNA-templated; P:regulation of transcription, DNA-templated

PtrGT52	6	F:chromatin binding; P:regulation of transcription, DNA-templated; P:post-translational protein modification; C:plasmodesma; P:positive regulation of transcription, DNA-templated; F:sequence-specific DNA binding transcription factor activity
PtrGT53	1	F:chromatin binding
PtrGT54	1	F:chromatin binding
PtrGT55	3	F:sequence-specific DNA binding transcription factor activity; P:post-translational protein modification; P:positive regulation of transcription, DNA-templated
PtrGT56	2	F:DNA binding; F:chromatin binding

P, F and C represent biosynthetic process, molecular function and cellular component, respectively.

Supplementary Table S7. Primers designed using Primer Premier 5.

Gene name	Sequences (5'-3')
PtrGT1	AATGCTGATGAGGACGGGGA
	CAGGGTCTTGAAAAATCGCTG
PtrGT2	AGTCACACTCAAACACCAC
	ACTTCATCGGCAAAACAAG
PtrGT3	CACCACAACATCAACAAC
	TGAGCAATCACAATAACT
PtrGT4	GGAGGATGGGTTGTTTTCGGA
	GCGGGCCTTTTATCATTTGGT
PtrGT5	TGGCTCTCCCGCTGCATCCTTC
	CCTCCTCGCTATCTGACTCTTG
PtrGT6	GGAAGAGGAAGGTTGAAAAA
	CTCCATTGACTGTCGCCATT
PtrGT7	TCGCGTGAAAACGTTAGGAG
	ACCATGAACATTAAAGCCCC
PtrGT8	CGGATTGCTTGCAGATTCT
	TCCTCCTCTTCACCGACTT
PtrGT9	GGTATTACTGCCACCTCC
	TCTCGTTCCTTCCTCTTC
PtrGT10	CATGTTTCCTCCTGATTT
	CTGATGTTTGTCCTCCTA
PtrGT11	TTTTCAACAAGCAACCCA
	AAGCCACCTCCCTCATCC
PtrGT12	ATTCCATCTTCTTTTCCA
	AACTTATTCTGCGTCTCC
PtrGT13	CCCTTCAGTTAGAAGAACGGAA
	GCCATCTGCTCATTCTCAAGTT
PtrGT14	ATGCGAGCCCAGACCCCTACC
	CCATCTCCCAATACCTTTACC
PtrGT15	CAGAAGCATTAACTATATACTCC
	AAACGCCTTTATTTGCCCCGCCA
PtrGT16	CTCTTTTGGTTTGCTTGC
	TCTTGATGAATTTTGGCT
PtrGT17	TGCTCTTCGGGCTAGGAAATATA
	TCCGTGATCCGGTAATCTAACAA
PtrGT18	AGATGTTTGAGCAGGAATG
	TTTCACTGATGAGTTTGTT
PtrGT19	TAAGAAACATCTCTCACC
	TAAAATAATCATCGTCCA
PtrGT20	ATGGGTTGTTTTCGGATTTT
	CCTTGAGCTTGACTTGCTTG
PtrGT21	CTTCTGCCAATCCTCCTTT
	TCTCCCTTTGCCCCCCTAT

PtrGT22	GAGATGGAGAAGACGAGGA<br>ACGATGGTATTGATGATGG
PtrGT23	AGATATGAATGGGTCAGGG<br>GAGAACAAAGGTGGGAATG
PtrGT24	GAGTTGGGTTACAAGAGGAGTGC<br>GTTTTTCGGTGCTACAAGCGTTC
PtrGT25	ATAGTAGGGGCATACATGGGGTA<br>TTCATATCAACTTGGGCAATCTG
PtrGT26	TCTTTTCCAAACTTCTCCCC<br>CTTTCATGTTCGCGTCTCTC
PtrGT27	TTCCACAACCCAAACTAACGCCAC<br>CTAATAAGAGCTTGAACTTCCACT
PtrGT28	CCCTAACGACTTCCCTCT<br>CTAACAAAACCCCACAGC
PtrGT29	AGAGAAGGATGAAGGAGATG<br>TAGAAGTGCTGTGATAAGGG
PtrGT30	GCGCTTCTTCTGCTAGCGTATCT<br>AACGTCATCACCTTTGGGTCAAT
PtrGT31	GGTTGAAGTGGGTGAGATT<br>TTGTTTGAAGGCAGTTGTT
PtrGT32	GCTCTGGTTCTGTTCTCAC<br>CTTTCTCTTCCTCCTTCGG
PtrGT33	AGCCTAAAATCCATCGTG<br>TGTTCTTTGAATCAGCAG
PtrGT34	GCCACTCAAAGGCACAACAAAAACC<br>AGCCATCTCTGCTGCTATCTCCTCC
PtrGT35	CTCTCAAAACTAAACCAG<br>CGACCTCCAAGAACAATA
PtrGT36	CGAAACAGCAGCAAATGATA<br>CACAACTGAACTGGAAGCAA
PtrGT37	GTGCCAGGCCAACACTCA<br>TATCACCCTCCCACCAAA
PtrGT38	GAGACCCCTGGGAATGGTGCTGAG<br>CATCCTCCAACCAGAATGCTCGCT
PtrGT39	TAACACAGACAAGAAACCCACC<br>GCAGCCCCATCAGATAAACCTA
PtrGT40	GTCTCAAAACTAAACCGG<br>TACGACCTCCAAGAACAA
PtrGT41	TATGATAGGAGTGTGGACGA<br>CAAATAAGAATTAGAAGGCG
PtrGT42	TATTGATTCTTATGCCCC<br>TACCCTAGATTCGTTACC
PtrGT43	CAAGCTATGTGGTAGAGAGACC<br>CAATCAATACTAATGTGGGAAA

PtrGT44	ATGGTGTGAAAGTGGATGTGTGGGG<br>GCAAGTGGTGATAACAATCGGCGGC
PtrGT45	AGGGATGTTGGAATCGGGT<br>AAGGTGCTTAATTGGTGAA
PtrGT46	TTCGGGTAATGCTGGTGGGTTTCG<br>GGCTTCAGGTTCGGGTTTGGGCTC
PtrGT47	CGGGTGGACAGGAAGCAA<br>TCTCAGAGTCGGAGGAAT
PtrGT48	CTATGTCTTGGATGGGAG<br>GATTTTGTTTTTTGGTTG
PtrGT49	TCAGATAACTCCATTCCCGC<br>TGTCCACTAACCAAACCCCT
PtrGT50	AAGTTGGGTTGCCTTTGG<br>TTTATTGCCCTTTTTGCC
PtrGT51	ATCTTCTGACGATGAGGA<br>CATTTGAATCTGTTGCTT
PtrGT52	GGCTGACTTGCTTGATGTA<br>GTTGCCAATTTGAGGTTTC
PtrGT53	TCTCATTAACTATCCTTCG<br>GCACCTTTTATGTTCTCAC
PtrGT54	CACGGTGAAGAAAGTGATG<br>CTATTTGAAGTTGAAGCCC
PtrGT55	TATTTGGATCGTGCCAATATGAAT<br>CATCTGCTCCCTCTTACTGCTCTC
PtrGT56	AAGAAAATGGACCAAAAGGG<br>AGAGCATCAAGCTGCTGAAA
Actin	AGGTTATGCCCTTCCACACG<br>TCGAGGGCAACATACGCAAG
RD29A	TTTGAAAATACCAGTAGGAT<br>AGAGTAAAGAATTGGCGTTA
RAB18	CTGGACAAGAGAGGTTTAGA<br>TCGTCGTGTTACATCATACA
PR1	TCCCTAATCCTTCCCTCTCG<br>AGGCTTGCACTGTGGTGTCC
PR4	CAGACCTGCCCTAATAACCT<br>TTGCTTTGACAGTTCTTGGA
5' GSP	CGGAACAACAAATAGCACCACTG
5' Inner	CAACCTTAGCATCCCTGCTGAGA
5' Outer	TGGCATCTTCTGTCCGTTTC
miR172d	GGAATCTTGATGATGCTGCAT
5.8s	GTCTGCCTGGGTGTCACGCAA

Supplementary Data S1. Information for trihelix genes.

>PtrGT1
MDNSGLGGRFLSGPNSGLLDLESPIHRHQQSQLGHPSLAHQHQVNLVGCFDNDHQPIGLSEVK
GTPSKVYSANFGKGKAVSPFNCASSGNASEDDDQSFMEDGNGENSTGVKGKKGSPWQRMK
WTDNVVRLLIAVVACVGDDGTLNAVEGLKRKSGLLQKKGKWKMVSKLMISKGCHVSPQQCE
DKFNDLNKRYKRLNEILGRGTTCRVVENPVLMDSMPHLSAKAKDDVRKILGSKHLFYKEMCA
YHNGQRIPNCQDLDLQGCSLPLERSSKDNNGSGEDEAEGNGDSDDGDDDDDESDNEENNNA
DEDGERVGQLCEGRVNDEHAHLWSQSGGRNGFDVEMAAIFQDPAVSPWERKEWIKKQRLQL
LEQRVSIQAQTFELEKQRFKWLRYCSKKDKEFERLRLENEWRKLENEQSALQLRQKQLEMDF
RSSEPSFDPACLGIDGVQGRDPIDLSRPQ
>PtrGT2
MDDDDDIQSHSNTTESSSPSPSPPDGRITVTVPATATVPPTPPPQQTDNSLALVLPMKSKANGSG
GGGREDCWSEGATAVLIDAWGERYLELSRGNLKQKHWKDVAEIVSSREDYSKSAKTDIQCKN
RIDTVKKKYKLEKAKMASGGGVSSWPFFDPLDRLIGSTARVPAVASGSKFPYHFRRNQNMKAR
IRKWGKDEDDDDDDEDEEEEEEEEEEEEEEEEDGGWGNSIRMLTQAMLKFGEAYEQAETAKLQ
QVVEMEKTRMKFAKELELQRMQFFMQTQMEISQLKNGRKGGSASGNHHHHHHHHHTGNNIT
ASNNVNNSDSDN
>PtrGT3
MDKETNQECPSLRPNNSTTIKEESPRKIPRGLGAAGVIVTGTGGGGGGGGDRLKRDEWSEGAV
STLLEAYESKWILRNRAKLKGHDWEDVARHVSSRANCTKSPKTQTQCKNKIESMKKRYRSES
ATADASSWPLYPRLDLLLRGNSATAAVISSPQHQQPAIPASSNPPLILLVDPTLGVPQPPPPSMTPP
PPPPPSPPPQVIVIAQNSHGSNGVDRGQKEDGVDTKLSNHVSDKNAMEVTDSSTPALYSDKKK
TRSKKLKMRKERRKWGKREEWEIADSIRWLAEVVVRSEQARMDTMREVEKMRIEAEAKRGE
MDLKRTEIIAKTQLEIAKLFAGGGKGVDSSLRIGRN
>PtrGT4
MALEQQLSLTPTQNPVDGERTNGVDTNIRDGGDDGNKAPRLPRWTRQEILVLIQGKRVAENRV
RRGRASGMGIGSGQIEPKWASVSSYCKRHGVNRGPVQCRKRWSNLAGDFKKIKEWETSIREET
ESFWVMRNDLRRERKLPGFFDREVYDILDGGGGTVPGLALALAPSSTAAEAEAVAEEVVFDSG
RSAAAEDGLFSDFEQEEGGGSPEAVVKEVQPIKMAVTAGVANPTPISEKQYQPAPRASQAQVPP
NDKRPATNPEMGSASHEERKRKRFVIDGDEETISLQSHLIDVLERNGKMLTAQLEAQNTNFQLD
REQRKDHADGLVAVLNKLANALGKIADKL
>PtrGT5
MEDLIESSSVTPSTAPHSRSLPIREDCWSEEATSTLVDAWGRRYLELNRGNLRQKDWQDVADT
VNALHGHTKKTHRTDVQCKNRIDTIKKKYKIEKSRVVSSNGTLTSSWPFFERLDALIGSNFNSSI
KKPPSPSLSPSPPVALPLHPSYRRTPPVSSTPPPQPAALAVALPQKRTLPVDDGGYFRRNYSAMA
AAAAAQESDSEEEEEEEEEEEFEAGERESVEEDGEGEGIKRLARAVERFGEVYERVESEKLKQ
MVDLEKQRMKFAKDLEMERMRIFTETQIQLEKIKKGKRAPDNERDY
>PtrGT6
MMFSGGGEGDGLGRINMMPTAAMLSPTAEISPRGPPQQQPQWGQQETKEFIGIRAELEKDFTV
TKRNKTLWEIVSVKMREKGYRRTPEQCKCKWKNLVNRYKGKETSDPETGRQCPFFEELHAVF
TERAKNMQRLLLESEAGSTQSRKKMKRTSGDRSSDEFSEEEDEDEDDSEEEKPVRSNSRKRKV
EKIIAEKSPRASSSTVGGIQEMLKEFLQQQQKMEMQWREMMERRSHERQMFEQEWRQSMEK
LERERLMIEQAWREREEQRRIREESRAERRDALLTTLLNKLIRENNI
>PtrGT7

METTPCATPKRVPPPCWTRDESLALIKAYCDKWYSVNRGNLRAADWEAVAVAVPPKSSLQCRH
KIEKLRKRYRVEKQKCLEYPGQFFSSWDLFPLLDSLEIGSLGSKGDQEIDKGNDVGDGFRVKTL
GDRYLLTTQKNGKTNGDLDPDEDFGLDPDLAFRAAKYSKVHGGFNVHGDSESGFGFKSVANK
NLVSVAFGPKDYVGVNGKLTANVGLNCDNGGEFGYKVEKTRGGRKVMPQGVRLPDYEMIAN
NYGSYVRDFTKGVHSYGGFPLKSLGDGNYNTIDGKPTPDFCDNDDHVEIDYRNDKRDEFGKK
VTNGWSYSPTGFRPKNCGTIDGNSRSNARSRAVNGSVNVGMNGIGDGVKRETDPISLLVSAIE
QATESFVKVEMMKMDMASEIEKMRMEMGLKHNQMILESQQRIVDTLAKAMLEKKKEREKV
EVRPPNSSRNGFSQVAAVEFERIGKSIVKEGEACFT

>PtrGT8

MDDTEDDARYPQNPYGENNQRSYGSNRHKLPVGNATYSRPVDNQYAVSEDEDSDDEEEGEHE
GIDDDHEDDDIERHPKKRKLKSLVSSYEFAPLGGRNPLTDWTEHETFVLLDAWGEKFLQRGKK
SLRSDEWQEVAEKVSDKSKIERTDTQCRNRLDTLKKKYKIERIKLAEDGGGASKWVYFKKMD
VLMSTSAQQGGLSCGMDSGEYVSMNPRVYSNHSNGFDEMRDSPGNSELARDEDDSDGLPPK
KRRLGRDCIEQSPFGLLADSIHKFSEIYEKIESSKRQQMLELEKMRMDFQRDLEMQKRQIIERA
QAAIAKIHQVGEEEDSISANSA

>PtrGT9

MEASTTFPENSNAATGNRDSDEGDEEMRVKAEEGDQHSTGNRWPKQETLALLKIRSDMDVAF
KDSGLKAPLWEEVSKKLNELGYNRSAKKCKEKFENIYKYHRRTKEGRSGRPNGKTYRFFEQL
QALDNTEVLLPPPSSDKVHTSMAAALVNPVSFIPNAVPCSIQSPGMNFVDTTSTSTASTSSEEEE
GTRKKKQKLTGFFERLMKEVIEKQENLQNKFLEAIEKCEQERIAREEAWKMQELDRIKREREL
LVRERAIAAAKDAAVLAFLQKFSEQGISVQLPDNPIVPMKFPDNQTVPVPSSAPVQLPKNQAVP
VENIVKTRENSSIESFVNISPSRWPKEEIEALIGLRTKLEFQYEENGPKGPLWEEISASMKKLGYD
RSAKRCKEKWENMNKYFKRVKESNKRRPGDSKTCPYFQQLDALYREKNRRVDGSGFELKPEE
LLMHMMGGQGDQQQPESATTEDRESENVDQNQENYRDKEDGDGDRIVANDPSSMEIME

>PtrGT10

MQQGGGERGSSQSQYAVPQQQQQQGDMPLPPSTSAALATHMQQQQQVVEEASPISSRPPATAA
TTSGGGVMNLDEFMRLSGGGGGAEEDIAGEDADRTGGIASGNRWPRQETLALLQIRSEMDAA
FRDATLKGPLWEDVSRKLAEMGYKRSAKKCKEKFENVHKYYKRTKEGRAGRQDGKSYRFFS
QLEALQNTGGGGGVSSSISNVSGVAPQLIGTATTSSLDVAPVSVGIPMPIRTPPPSSQVPQPASNIG
SMFPPDLGATVARAAAAGAPVRISFSSNESSSSQSSEDDDDDEDEGILGGQTSAMGAGTSRKRK
RASLSSSKGETHRMMEFFEGLMKQVMQKQEAMQHRFLEAIEKREQDRMIRDEAWKRQEMA
RLSREHEIMAQERSISASRDAAIVAFLQKITGQTIHLPTPVSIAPLVSQPQPPPPTQPQQVQIAPLV
TVSTQPPLQPQPMPLSQVTPQQNKQLPQQQHHQQQQHQQVHHQHQPPSISSEIVMAVPEQQIA
PLELGSGGSEPASSRWPKPEVLALIKLRSGLETRYQEAGPKGPLWEEISAGMLRLGYKRSSKRC
KEKWENINKYFKKVKESNKKRTEDAKTCPYFHELDALYRKKILGSSSGGAGSTSTSGFDSQIN
RPQKQQHQHQESLELDPMPPPMQQTVPQQTQATESQNKNGASVDVQASNTVLAGSPFGEGN
GGAEKKPEDIVKELMKQQGTQQQQQLMVDDYDKMEEGDSENVNEDEYDEEDDGDEDEEED
EALQEERKMAYKIEFQRQNTSNATNGGGSGAPSFLAMVQ

>PtrGT11

MIGDSSVQATSSDVAATATRVATEGGEGGGGGGFGSNSAEEDKTMGVDHEGNRMNYGANRW
PRQETLALLKIRSDMDAVFRDSGLKGPLWEEVSRKLAELGYHRSAKKCKEKFENVYKYHKRT
KEGRTGKSEGKSYKFFDELEAFQNHPPHSTQPPTLTPPPLPPPKAQTASATITTLPWTNNNTAIVS
HATVPSRTNPMDIMSQSIATPTNNRAISPMPISSNPINPSQNAYPSSLQNLTTHLLASSSPSSTASD
EELEVSYKKRKRESNWKDFFERLTRDVIKKQEDLQEKFLETIEKYEHERMAREEAWRMQEMA

RINREHETLIQERSTAAAKDAAVVAFLQKISGQQNSVQTQEIPQPTTTPTAPPSQPLQLRPPPSLA
PVAKLEVPKRDNGDNFTVSSSSRWPKVEVQALINLRANLDVKYQENGAKGPLWEDISAGMQK
LGYNRSAKRCKEKWENINKYFKKVKESNKKRPEDSKTCPYFDQLDALYKEKNKMEITVNSDY
AVKPTSTMEPLMVRPEQQWPFQQATQPQTIIEDNERNINIDHNIEDDDDDDVVVDDDDVDTDE
EDEGGGFEVVANKSAPLVNGDQ
>PtrGT12
MLGDSSSVLATTTTTLAASGGGGVGNREAPPLTGGHHEGRNIDVGGEDDKGVIEGHRGIGGNR
WPRQETMALLKIRSDMDVAFRDASVKGPLWEDVSRKLAELGYNRSAKKCKEKFENVYKYHK
RTKDGRSGKQEGKTYRFFDQLEAFQSHPPSLSSPLPPQPTKPHIPPANTIAMPVVNPSPNVVGTS
RNTVPSTAAATLATNTSQGIVTSAINLAVPPFPSTDPTICPPSQATNPTNHPRTNIPSSFPNFSSDLN
SNSTSSSTSSDVELQGRRKRKRKWKDFFERLMTEVIQKQEETQNKFLEAIAKRENERMVREES
WRMQEMARINREREISAQERSIAATKDAAVMAFLQKLSEQQNPGQVQNNPPPTQPPPPALPPIS
QQTPTPTPPPPLPVAQVPPPQPVANLDIMKSDNGDQNFMSASSSRWPKVEVEALIGLRTNLDCK
YQENGPKGPLWEEISAGMRKLGYNRNAKRCKEKWENINKYFKKVKESSKKRPEDSKTCPYFH
QLDALYKEKNKIDGPSNSTHHMKPQNSVPLMVLPEQQWPPAQQEHRPDSMMGDLESDDNQN
QEDGDDKFMDDEDEDEASGYEIVANKQTSMNTAG
>PtrGT13
MEGNLSQGGMIPGGAPFGGLDLQGSMRVHHQAQHPHTMHHHQHPLHRQGSSTLTSVEEGFPL
TMGFMHNSDQNISMTDYNKGDRGKNSVSDEDEPSYTEEGADGHNDAITGKKGTPWQRVKW
TDKMVRLLITAVSYIGEDGTSDCGGGMRRKFTVLQKKGKWKSVSKVMAERGFHVSPQQCED
KFNDLNKRYKRLNDMLGRGTSCQVVENPALLDVIDYLTEKEKDDVRKILNSKHLFYEEMCSY
HNGNRLHLPHDPALQRSLQLALRSRDDHDNDDARRHQHDDLDEDDQEIETDDHDEFEENHAS
HGDCRGIHGVLGGSAKRPRQGQGHEDAFSPESSKAVWLQKQWMESRTLQLEERKLQIQQEML
ELEKQRFKWQRFSKKRDRELEKLRMENERIKLENEQMALELKRKEMGADFN
>PtrGT14
MSAPIKTGSPSSPQDLTPLQTTAQQTLPAPPTHPSTNPRRLPPPCWTPDETVALIDAYRDKWYTL
RRGNLKANHWQEVADAVARRCPDASPPKTAVQCRHKMEKLRKRYRTEIQRARSMPVSRFTSS
WVHFKRMDAMEKGPQAKADYNSESEGDDNDDDNEDDGDIRGSYLENCRNAKNFMNTRSIQ
KLYGNGIGNPGNGGDNNGLSGGNSSLGSGNAGGFRIRIPTGVSVAQPGPKFYAKTEQKHGGSP
NMGVNASPDPYPKPKYGGGVGTSSRVMRGSEEMGRKREREPMEELVAAVKVLGDGFVRMEQ
MKMEMAREMETMRMEMEMKRTEMILDSQQRIVEAFAKALSEKKKRPKRMPSPES
>PtrGT15
MSTPSPSPSPPPPHSPPTNPTPLSSKKTQPLPWTHQETIHLIQAYQEKWYSLKRGQLKASQWEEV
AVTVAARCGYDYNHPSKSAVQCRHKMEKLRRRYRDEKRVMALGGTCYWQYFDLMDSLERG
PLPISAQPLARVPCQENYHTRNRNNGVLGEYDDDKGEDEDDDDDEDYGYRSKLRSRSINYILR
KPSIVNRYAGGGPSGVSREAEKKRKREEVEEEEEEEEEEEEGEGVEMGLAGQIKAFSERMVKL
ERKKLEVMKETERSRMEMENKKLEMILDSHSKIVHMIGEAFGL
>PtrGT16
MDDTEDDARYPPNPYGENNQKSYGSNRHKLLAGNAMYLREVDDQYSIDEDEDSDEEEEDEH
ELGVEDGENNQKTGIHYEGKDVDEEEEEEEEGVDDEEDEDDNQKYNRRIDEDEDDNEKYNM
RIDDDDDDGDIERHPKKQKLKSLVSSYEFAPRVPAPHVAVPAGPKPSVGGRNPLTDWTERETFV
LLDAWGDKFLQRGKKSLRSDEWQEVAEKVSEKSKIERTDTQCRNRLDTLKKKYKVEKIKLAE
AGGGASKWVYFKKLDILMSTSAQQGGLSCGLDSGEYVFMNSKAYSNHANGLDGMRDSPGNS
ESAHDDDDSDGLPPKKRRLGRESRNCNEQSSFGLLADSIQKFSEIYEKIESSKRQQMLELEKMK

MDFQRDLEMEKRQIIERAKAEIAKIHQDSEDEDTMSANSA
>PtrGT17
MSTAPSSDFTTKRVPPPCWTQEESLALIKAYRDKWYSVNRGNLRVADWEAVAAAVPLKSSLQC
RHKIEKLRKRYRAEKQKCLKHPGRFFSSWDLFPLLDSMEIGSLGSKVEQEIDKGNDAGDGICV
KTLGDRYLLTAQKNGKINGDLDPDEVFDLDPDFALRARKYSKVDGGFSVSKDSGSGFGVSSVA
FRPNDYVGVNGNIKANVGFNCDHGGEVVRLPDHGMMADHYGSYNSDFSKGVDVYEGFPLK
SLGDRNLPMQGLNPKNYKKIDRKPTPDFCDDDHVEIDYRIDKRDRFGKKVNDGWSSSFPGFIP
NNCGNIDGKSRSDSRSRALKGSVNVGTNGIGDGVRREADSVALLVSAIEQATESFVKVEMMK
MDMAMEIEKMRMEMGLKHNQMILESQQQIVDALAKAMLEKKKRRKVEVLSSNSNRNGYTQ
VAAVDCESIGKSSIVKESEACVS
>PtrGT18
MMFRSGGGEGDAGLGWINMMPTAAMLSPTAEISPRGPAPPQHPQWGLQETKEFIGIRAGLEKD
FAVTKRNETLWEIVSAKMREKGYYRRTPEQCKCKWKNLVSLYKGKETSDPETGRQCPFFEELH
EVFTERAKNMQRLLLESEAGSTQSRKKVKRINGDRSSDELSEDEDEDESEEEKPARNNSRKRK
GEKNVAEKSPRPSSSSFGGVQEMLKEFFQQQQKMEMQWRELMEKRAHERQMFEQEWRRSM
DKLERERLMIEQAWREREEQRRIREESRAERRDALLTALLNKLISENTMKD
>PtrGT19
MGDLIESSSVTPSTTPHSRPLPIREDCWSEEATSTLVDAWGRRYLELNRGNLRQKDWQDVADA
VNALHGHTKKTYRTDVQCKNRIDTIKKKYKIEKSHVVSSNGTLTSSWPFFERLDALIGSNFNSS
GKKHLSPSPPVALPLPPSYRRTPQVSSTPPPQPPALAVALPQKRPLPVDDDYFRRNYSAMAAAA
AAVESDSEEDEDEEFEGGERERAEEDVEGEGIKRLALAIERFGEVYERVESEKLKQMVDLEKQ
RMKFAKDLEMERMRIFTETQVQLEKIKKGKRAPEDY
>PtrGT20
MALEQQLNLTPTQNSVDGERTNGVDTSIRDGGDDGNKAPRLPRWTRQEILVLIQGKRVAENRV
RRGRASGMGIGPGQVEPKWASVSTYCKKHGVNRGPVQCRKRWSNLAGDYKKIKEWEASIRD
ETESFWFMRNDLRREKKLPGFFDREVYDILDGGGGTVQGLALALAPSSAAVEAETIAEGVVFD
SGRSAAAEDGLFSDFEQEEGGRSPEAVVKELQPIKAAVAIPTPISEKQYQPAPQASQAQGSLNDK
RPSTNPEMGSASHEDRKRKRFAIDGGEETISSHSHLIHVLERNGKMLTAQLEAQNTNFQLDREQ
RKDHADGLVAVLNKLADALGKIADKL
>PtrGT21
MDKETNRESPSLLPSNTTTIKEESPRKLLGGTGAAGVVVSGGGGGGGDRLKRDEWSEGAVSTL
LEAYENKWILRNRAKLKGHDWEDVAHHVSSRANCTKSPKTQTQCKNKIESMKKRYRSESSTA
DASSWPLYPRLDLLLRGNSATAAVTPSPQVPPQHQQPSIPTSANPPLMLLMDQSPLAVTQPPAPA
PPPPPLPPPLPHQTIGTAQNSHGSNGVDRGAKGDGMDTKLSDHVSDKNAMDTDSSTPALYSDK
EKLRSKKLKKKMEKRKRRKREEWEMADSIRWLAEVVVRSEQARMDTMRELEKMRIEAEAK
RGEMDLKRTEIIANTQLEIAKLFAGVGRGVDSSLRIGKT
>PtrGT22
MEDDDEIQSHSNTTEYSSPSPPPPNGRITVSAPAAVHPQPAPPPPQQNNKNRLALVLPTKPKVNG
GGGGGGGREDCWSEGATAVLIDAWGERYLELSRGNLKQKHWKEVAEIVSSREDYSKSSKTDIQ
CKNRIDTVKKKYKLEKAKIASGGGPSGWPFFDPLDRLIGSTARIPVVGNGNVGSKIPTRVRSGS
RRGGVNQYHFRNQNVKIRILKHEDDEDDEEEGEEEEGGAESDDSFPPMKKRRVVVEREVRGK
VGKEKRGGGWGNSIRMLTQAMVKFGEAYEQAESAKLQQVVEMEKTRMEFVKELELQRMQF
FMQTQMGISQLKNARRGGNATSNHYHHQYHRHHTGNNINASNNVNNSDSDN
>PtrGT23

MDNSGLRGRFLSDSSGGLLDLESPIHRHQQSQLGHPSLAHQHQMNVMGCFDNDHQPSGLME
MKGSTSKGYLVNFGKGRAASPFNGAHSGDGSEDDDQSFMEDGNGENSTGAKGKQGSPWQR
MKWTDNIVRLLISVVACVGDDDTFDGTGGLKRKSGLLQKKGKWKTVSKLMIGKGCHVSPQQ
CEDKFNDLNKRYKRLNEILGRGTSCRVVENPALMDSMPHLSAKAKDDVRKILSSKHLFYKEIC
AYHNGQRIPNCQDFDLQGCSLPLERCSKDMNGSGGDEVEGNDDSDDDESNNEADNNADENG
ESVGQLCERIVNEEHSHLCSQSGRQNSFGVEMTAIFQDTNVSPWERKEWIKKQRLQLLEQRVN
IKAQAFELEKQQFKWLRYCSKKDKEFERLRLENEMMRLENRQSAFQLRQKQLEMGLRSSEPA
YDPTSLGIDRVQGRDQIDLGRHH

>PtrGT24

MFEGVADQFHQFIASRTTSLPLPSLSFPPLHGSSNTTNFPSFYPYTATAATTSQVPLQPNLLHSLH
QGSPTNKNDEKQENNLMAMNLKFERERSIPELVNPWSNDEVLPLLRIRSSMDNWFPEFTWEH
ASSRNLAEVGFKRSTEKWKEKFEEESGYFNSNIDIYSKNYRASFSEFEEIYHGDQNPDQQEATA
GEKKIRKPSEDEQQDKMGQNLEEETRIDQTVGNQSVEDNDGKLEQFEKSKRKKRKREKKFEM
FKGICEDIVNKMMAQQEEKHNKLLEDIVKRDEEKFAREEAWKKLEMDRINKELELRAHEQAL
AGDRLDTLIKFLKKITSGDSYAEILGETSAPDVVKVPKCSNASTSSSLLLAQNPNPASQTSCESQ
LETPTSSTTTLDHQKSTSMPAKSNTSCTENQEPQNPNSTLAPNIPQAPTTSSTLALAPQNPNSLN
SHNSPSGPSSILPMYKVQAKSTSNDEDDIGKRWPRDEVLALINLRCSLYNNNEDKEGSAKAPV
WERISQGMLELGYKRSAKRCKQKWENINKYFRKTKDASKKRYINSRTSPYFHQLSTLYNHGTL
VAPKNRSASPENQSNLSETRHSSSSSQNGTSNSAMHVVEGEKNKVQVPPFDFEF

>PtrGT25

MIPGGAPFGGPDLQGSMRVHHQGQHPHIVHHHQHPIHRQGSSTHPSVHDGFPLTMGAMHKSD
QNMSMTDYNKGDGRKNSVSDEDEPSYTEEGADGHSDVRRGKKGTPWHRVKWTDKMVRLLI
TAVSYIGEDATSDCGGGMRRRFTVLQKKGKWKSVSKVMAERGFHVSPQQCEDKFNDLNKRY
KRLNDMLGRGTSCQVVENPALLDVIDYLTEKDKDDVRKILSSKHLFYEEMCSYHNGNRLHLP
HDPALQRSLQLALRSRDDHDNDDVRRNQLDDLDEDDQEIETDDHDEFGENHASHGDSRGIHG
VLGGSAKRPRPGQGHEDVYFGNSSQDPNKGSSYSHPQIAQVDMNQVSPESSRASWLQKQWM
ESRTLELEEQNLKIQQEMLELEKQRFKWQRFSKKRDRELEKLRMENERMKLENERMTLELKR
KEMGADFN

>PtrGT26

MLGDSSSVLATTTTSTPGGGGGGGGAGDHKEAPPLTGGNHEGGSNDVGGEEDKGKIEGDRSY
GGSRWPRQETLALLKIRSGMDVAFRDASVKGPLWEEVSRKLAELGYNRSGKKCKEKFENVYK
YHKRTKDGRTGKQEGKTYRFFDQLEAFESHPPSLSSPLSLPPQPPKAPTPAVTAIAMPVVNPSPN
IVRASHSTVPSTAAATLATNMSQGIVTSAIIYLTVPPFPSTNPTILPPSQATNPTNPPHTNIPPSFPN
FSPDLISNSTSSSTSSDVELQERRKRKRKWKDFFERLMKEVIQKQEEMQKKFLEAIERREHERM
VREESWRMQEMTRINREREILAQERSVAASKDAAVMAFLQKLSEEQNPGQIQNNPPPSQPPRPP
APPPILQLVPTPPPPLPQAVANVDMIMKSDNGDQNFTSASPSTSASPSRWPKVEVEALIRIRTNLD
CKYQDNGPKGPLWEEISARMRKLGYNRNAKRCKEKWENINKYFKKVKESKKKRPEDSKTCP
YFQQLDALYKEKNKIDGPSNMKPENSVPLMVRPEQQWPPPQQEHRPDSEMEDLESDDHQNHD
DEDDKNMDDEDEEEDEASGYEVVANKQTSMNTDE

>PtrGT27

MLGDSSVLASSSDVATTATRVVPEGGEVCEVGDAGGFGSNSTEEDKNMGGDHEGDRMNYGA
NRWPRQETLALLKIRSAMDAVFRDSSLKGPLWEEVSRKLAELGYHRSAKKCKEKFENLYKYH
KRTKEGRTGKSEGKTYKFFDELEAFQNHHSHSAQPPTILAPPLPPPKAQTPTATTATLPWTNSPA
IVSHVTVQSTTNPIDILSQGIATPTTIHSTISPMPLSSNSLNPSQDTLPSSLQNLATHLFSSSTSSSTA

SDEKLEGSRKRKRKRNWKDFFLRLTRDVIKKQEDLQKKFLETVEKCEHERMAREDAWRMKE
MARMNRQHEILIQERSTAAAKDAAVFAFLQKISGQQNSTETQAIPQPKLTPPPTQPPQPRPPPTS
LEPVTNLVVSKWDNGENVTVSSSSRWPKVEVQALISLRADLDIKYQEHGAKGPLWEDISAGM
QKLGYNRSAKRCKEKWENINKYFKKVKESNRKRPGDSKTCPYFDQLDALYKEKNKMESRVS
TGYAVKPISTMEPLMVSPEQQCPFEQANQPETIIEDNERDINIDHNIEEDDDDMDDDTEEEDEG
VGFEVVANRPASLTNGE

>PtrGT28

MDLLAADDRQIRNPNDFPLHLIPFPPTGNFDISTSAAAVVGSSDVDSLLPQKLRPIRGNGRVPSG
SGEDGREKLWGFVSGEASDVAVNGGGGLGLCSCDGNADDSSVGAGESVGRKRKRRSKRKIEK
FLESLVMKVMEKQEEMHKQLVEMIESRERETMIREEAWKQQEMERMERDNEARAQETSRNL
ALISFIQNMTGHLQVTGSKGTNIWDAISDGMCNMGYNSTAKNCKEKWENINKHFKKSVGSVV
KNPFENITVNPYFHELDVLYNNGFVNLGNGSTNTGKQANCTLGKG

>PtrGT29

MEGLHHHLHHHQQQIQQQHHISVNVDTSDRFPQWSIQETKEFLMIRAELDPTFMEKKRNKVL
WEVISKNMKEKGYNRSAEQCKCKWKNLVTRYKGYETIEPESMRHQFPFYNELQAIFTSRMQR
MLWVDAEGAASGSKKKAAQLSSDEEEDNEEISEGEKGSSRKRIKKGKAVGGASCSTGNSNSLR
ETLEDFMKQQMQMEMQWRETFEARENERRMKEMEWRQIMEGIENERTTMDRRWREIEEQRR
VREEARAEKRDALITALLNKLRREDM

>PtrGT30

MASSCDEDFSLLGDDNHHHHIIHHPYRYPTKSTPIHAPPESVLPLDGEEDRQDNNETDSAFADV
NPYSDNETSKRSEREGEIDGGETPYNIYKRGRSGGGEYRKDREEWSDVAIGCLLDAYMEKFTQ
LSRGNLRGRDWEEVAAMVKQGKSVEQCKNKVDNLKKRYKLERHRITTGSISTSHWPWFQKM
EDIVGNSLTIKVAPPAPAPADEDNGGGGASSASVSRQSKRYANTIASPAGQTNNMMPKSISKTR
WRRVILKISGVALAGTGPNNIDPKVMTLIAREVAMACQLGLEIAIVVGARNFFCGNTWVTATGS
HRRTAYQIGMMASVMNSLLLQSALEKTGIQTRVQTAFAMQEVSEPYSRQRAIRHLEKGRVVIF
GGIGAGTGNPLFSTDTAAAVQASEIHAEAVLKGTNVDGVYDCHSPDSNFIFEHISIRDLVSRGAT
SMDMTALTFCEENNIPVVVFNLLEPGNISKALCGEQVGTLIDQTGRIG

>PtrGT31

MGSGILGLELPLHQQQNPQNPHHLQHPQIVAYAHHESDHHQHSQQSAKHAYPFASTKPKQLSP
LSNEDEPSFTGDDSTTDGNRKISPWQRMKWTDNMVRLLIMAVFYIGDEVGSEVSDPIGKKKPG
GLAQKKGKWKSVSRAMMEKGFYVSPQQCEDKFNDLNKRYKRVNDILGKGTACRVVENQSLL
DTMDLSPKMKDEVRKLLNSKHLFFREMCAYHNSCGHAGVASGINNSPEMATDPSHAQHQQAT
QQQQQYQRCSHSTENAQVVTNSRTETEGSKMAKMARNEEDDHDESEEDEDDYVEDEEEEDD
DDERDDDEEDYEENEDEAVEGNSRHQNGHGHGDEDDHDEKSSRKRPRKEVFSATSSLMQQL
NGEIMNVLRDGAKSSWEKNQWMKLKLMQLEEQQVNYQCQAFELEKQRLKWVRFSSKKERE
MERAKLENERKRLENERMVLTVRKKELELLDTTHQQQLPSNKRSDPSSIAG

>PtrGT32

MQPSYGLPNIDHHQQQQQRQQHLMEDDYSCSPSVLPISNLQNLNYPYQPHLHHYLQQHKHP
HQVLLQQHHQLFYPFQQQQQVQGQSQDQVNPSLFSVNFKWGRDYEDSGDKKAGALNNQEE
AACTTTNTTTFLDGNVHSNPPHAILMPHSWHPREDSTTLKEPFWKQLNKQRSGSEEKEINESK
TSYFSPLEMEQVVDESSEGCKNLESKFPLFNELQAIYSLAQIAEANQTGSGSVLTCDNSPTNAG
LSMPCNASNGQNVGACGAANNVIGVDRGSENSIGEEASLRKCRRRKRKGKLKEKLNYIAGFF
ENSVKKVMNHQEMLHRKFLEVIERMDKERAEREATWRRQEAEKYSREAISLAHERASASSRE
AQIISYIEKITGQSINLLTRMAPPLLQPEISNEPIKEITPTKTDSHSKWPKDEVEALIQVRSRIEIKF

QEPGLKGPLWEEVSSLMSSMGYQRSAKRCKEKWENINKYFRKARESPKRGSARSKTCSYFNQ
LDQLYSGTLINYPANTTDMPSSGIEFGIKKQGYTELFEVFSVRRDYLANVRNPLGVSVISSEMGS
SRSEFDGFSDQNTELEEGSSEQDIEAYKDDKQKDGEQQNGGGGGDEEIEN
>PtrGT33
MEVIVNKKKRRKEQRESKNNNQLRVSSSTPRTRSQVSPEWTTKEALILVNEIAAVEKDCLKALS
TYQKWKIIVDNCVVLDVARNLNQCRTKWNSLVNEYNLIKNWDKESESRSDFYWSLESERRRE
FGLPENFNDELFRAIDDYMWCHKEHPDTDPDPDPDPDTDSEKPDLLHAITNPGTKKQRWRSKS
LRTPMEEKTHKCCREDNSQTIHAEEKPQERCVEENSQIYCTVEKPQRVHAEEEHQESQVQEST
QIYNAGGKLQTIHAEEDPQESCAEEEPHTIHAEEEPEESPAEEHPQPCCSKEKPRAIHLEEETQER
YLEENHQTCCTNEKPQSILAETQLQESHEEEKPQKCRRKENSQNAHGDEKPKIHRGRKKKMPS
TEEMKQMMVEKLHENAEMIQAVVNGNFPEMADLEAADSKNIEGFKTDLIRRQGDKLIACLQN
IVNSINQFPCLLQECD
>PtrGT34
MSEHHHQYGMPDLRQLVAGRSHFQGTQQGGEPFFVQSRNLAAPQTHHFESIMVGHEAMLSSG
LVKLGGHHDRYCTNATNINTTNSATIGTSSSAGVGTLYGVEMESATTAGWIGNDGGNNSRWPR
QETLTLLEIRSRLDSRFKEANQKGPLWDEVSRIMAEEHGYHRSGKKCREKFENLYKYYKKTKE
GKAGRQDGKHYRFFRQLEALYGEPSNQASASETHFVNNTLLYQAPMSNTINQESQETFQENKH
SESLSFSNTSEFETSSSENNDDDLSAIAYNMMNRSTEKQKGINESQSLARPKKSWKLKVKDFVD
SQMRKLMEKQDAWMEKMLKTIEDREHERMCREEEWTKQELARFDQEHEFWAKERAWIEAR
DAALMEALKKHTEKGLELSSSVEQIAVATQRHNKNPDSAVAKKIQKDKFNNITWTEPEILSFIQL
RTSMDSRFQENGYSNEGLWEEIAAEMASLGYDRSVDECKEKWESMNIYFNMTTESNKKRKE
DLRTSNYFQQLESYNGMNSSPSNSYVGSQVNDNSCFQVQINEGDQHLWNTNKFDLKLNKEKN
QQQLWHNK
>PtrGT35
MATDDFPLIDEPPPHPPTRHPYSVTTTGDYYYSDHDKSAATPSSKRNQQNHYYNNNKRSKSNSI
TEIESCDYRDYRKDREEWSDAAISCLLEAYTEKYNQLNRGNLRGRDWEEVAEAVSERGGSNK
KSVEQCKNKIDNLKKRYKVELQRISGSGSSWHWFKHIEAIMGNACNGKSGGGGESDGGGGG
GSGGNVNAAVKQVKRYTSGSAAFANTLKTKPVPNLKWRRVVFKISGSALAGNCQNIDPKVAM
QIAREVATASRGGLEIAIVLGGRNFFCGDSWISATGLERPTAYQISMMATVMNSILLQSALEKHG
VQARVQSAFAMPELAEPYSRQRAIRHLEKGRVVIFGGAGAGAGAGAGNPLFTTDTAAALRASE
SIPLLLAFIISCVLLFTGNLDACFFYNVFWLSVNADALLKGTTNVDGVYDNHAGNSNAILDHIS
FREVVSRGVTSMDMMAITYCEENGIPVVIFNLLEPGNISRALCGDQVGTLIDQAGRIS
>PtrGT36
MDPSPSAAITRPHHGGREDCWSDGATGTLVEAWGDRYINLNRGNLRQKDWKEVADAVNNRQ
NGVKPRKTDIQCKNRIDTLKKKYKIEKSKPPPSTWPFYYRLDSLLGTNSNATNTFKKPTSVTFT
VKSKTKPQNDVYPGLASCGESSSDDDDMAWFDERVKKKRHRMEDVDLSDGAACRELARAIL
KFGEIYERIESSKQQQMIELEKQRMEFTKEVEFERMNLFVDAQLELKKKSFSRDKFASSSVVLQ
V
>PtrGT37
MYLSEKPRPLDFYKEEVGPSSSRDNMIIEVVSSNGDLPPLHLHPITAATNPHQMILGESSGDDNH
EVKAPKKRAETWVQDETRSLIGFRREMDGLFNTSKSNKHLWEQISTKMRDKGFDRSPTMCTD
KWRNLLKEFKKAKHQDRGSGSAKMSYYKEIDEILRGRNKNSQYKCPTPKVDSYMQFSDKGF
EDTSISFGPVEASARPTLNLERRLDHDGHPLAITAADAVAASGVPPWNWRETPGNGAESQSFG
GRVISVKSGDYTRRIGIDGTTDAIREAIKSAFRLRTKRAFWLEDEDQIIRALDRDMPLGNYTLHL

DEGLAIKVCLYDQSDHIPMHTEEKIFYTEDDYRDFLSRRGWTCLREFDGYRNIDSMDDLRHDA
IYRGVS
>PtrGT38
MYSSEKPRPLDFYKEEVTPSSRDNMIIEVVSSNGDLPPHHLPTTTNTNPHQMILGDSSGDDNHE
VKAPKKRAETWVQDETRSLIGLRREMDGLFNTSKSNKHLWEQISAKMREKGFDRSPTMCTDK
WRNLLKEFKKAKHKDRGSGSAKMSYYKEIDEILRERNKNPQYKSPIPSKVDSYMQFADKGFE
DTSISFGPVEASPRPTLNLERRLDHDGHPLAITAADAVAASGVPAWNWRETPGNGAENQSYGG
RVISVKYGDYTRRIGVDGTADAIKEAIKSAFRLRSKRAFWLEDEDQIIRSLDRDMPPGNYTLHL
DEGLAIKVCLYDESEHMPVHTEEKIFYTEDDYREFLSRRGWTCLREFDGYRSIDSMDDLRHDA
IYRGVS
>PtrGT39
MAPPWLSRRLLERWRPYTLIEAWGDRYINPNRGNVGQKDWKEVADTVNNRQNGVKPKKTDV
QCKNRIVMLKKKYKIEKSKPPPLSWPLCNRLDSLIGTNSNTTNTDKKPTSFTVKSKKKPKKRM
FSGLASYSESSSDDDEDDMAWFEERLKKKRHRMEDVGLSDGAACRELARAILKFGEIPERIESS
RQRQMIELEKQRMEFTKEVEFERLNMFVDAQLDHTKKSFKRDKFTSSSVLPVVCRGRQEMWI
FRTDSLQLNYFDKFVVHTDIYDSWIFPYN
>PtrGT40
MSTDDFPLLDAPPPHPTTRHPDAASVTTTGDYYYSDHDKSSATPSSKRNHQNHYYNNNNKRS
KSNSNTELESSDYRDYRKDREEWSDTAISCLLEAYTEKFNQLNRGNLRGRDWEEVAEAVSERG
GSNNKKSVEQCKNKIDNLKKRYKVELQRISGSGSSWHWFKHIEVIMGNACNGKSSGGGAAAA
AGGDSEGGGGGGSCGNANVVVKQVKRYTSGSAAFANSLKTKPVTNLKWRRVVFKISGSALAG
NCQNIDPKVAMQIAQEVATASRVGLEIAIVLGGRNFFCGESWISATGLERPTACQIGMMATVMN
SVLLQSALEKHGVQARVQSAFAMPELAEPYSRQRAIRHLEKGRVVIFGGVGAGAGNPLFTTDT
AAALRASEINADALLKGTIVNGVYDSHAGSSNIILDHISFRDVVSRGATSMDMMAITYCEENGI
PVVVFNLLEPGNISRALCGDQVGTLIDQAGRIS
>PtrGT41
MSEHHHQYGLPDLRQLVAGRAHFQGTQQGNEPFFVQSRNLVPQTHHFESIMVGHEAMLPSGL
VKLGGRHHDHYCTNATNITTNNTITSATIGTSSSAGAGTLYGVEMENATAAGWIGNDGGNNSR
WPRQETLTLLEIRSRLDSRFKEANQKGPLWDEVSRIMAEEHGYQRSGKKCREKFENLYKYYKK
TKEGKAGRQDGKHYRFFRQLEALYGEPSNQAPASETHFANNTLLYQTPLSNTINQESQETFQEN
KHSESLSFSNTSEFETSSSENNDDDLSAIAYNMMNRSTEKQKGVNESQSLAGPKKSWRTKVED
FVDSQMRKLMEKQDAWMEKMLKTIEDREYERMCREEEWTKQELARFDREHEFWAKERAWI
ESRDSALMEALKKHAEKGPELSSSVEHIAVATQRHNNNQDSTSAKKIQKDKFNNIIWTEPEILSF
IQLRTSMESRFQESGYSNEGLWEEIAEEMASLGYDRSVDECKEKWESMNIYLNMTTESNKKR
KDQDLRTNDYFQLLESYNGMNSSPSNSYLGTQVNDNSCFQVQINEGDQQQHLWNTNKFDLK
LNKEKNQQVWHK
>PtrGT42
MQLGLLGGFSLYCTSSRKPLKTTSPNMAAAFSALSSCPYTFFCRPSSTKLCVSCSAGSPTTTTIG
SRGTKAPPRKRTGRKEGTGKSMEDSVKRKMEQFYEGPDGPPLRIVPIGGLGEIGMNCMLVGN
YDRYILIDAGVMFPDYDELGVQKIIPDTTFIRRWKHKIEAVIITHGHEDHIGALPWVVPALDHNT
PIYASSFTMELIKKRLKENGIFVPSRLKVFKTKRKFTAGPFEIEPIRVTHSIPDCCGLVLRCADGTI
LHTGDWKIDESPLDGKVFDRETLEELSKEGVTLMMSDSTNVLSPGRTISESVVADALLRRISAA
KGRIITTQFASNIHRLGSVKAAADLTGRKLVFVGMSLRTYLDAAWKDGKAPIDPSTLVKVEDID
SYAPKDLLIVTTGSQAEPRAALNLASYGSSHAFKLNEEDVILYSAKVIPGNESRVMKMMNRISE

IGSTIVMGKNELLHTSGHGYRGELEEVLKIVKPQHFLPIHGELLFLKEHELLGKSTGIQHTTVIK
NGEMLGVSHLRNRRVLSNGFVSLGKENLQLMYNDGDKAFGTSTELCIDERLKIASDGIVVVS
MEILRPQNLDGQVEKSLKGKIKITTRCLWLDKGKLLDALHKAAHAALSSCPVNCPLTHMERTV
SEMLRKMVRKYSGKRPEVIAIAVENPAAVLSDELNSRLSGNSHVGFGISALRKIVDGHPKGNQ
VDRKQPDGNGYAHLEKTSPQNLEVDGIEFERELPKEEGTSSSPNLAEGHSSASEDQDDFQKSSV
PSSSPVNELVKSDESLVPPGEQMNKLKEDVMDSSDDDLLENENSRLKRSKSVKRNKWKPEEV
KSLIKMRGELHSRFQVVRGRMALWEEISTNLMADGINRSPGQCKSLWTSLVQKYEESKNGKK
GKKAWPYFEDMDNILSDSETMATK

>PtrGT43

MMADQGGKSLIREYKKGNWTVSETMVLIEAKRMDDERRMKRSDSAEGRSKPTELRWKWVE
DYCWKQECLRSQNQCNDKWDNLMRDYKKVRDYERKIAETGERNGGSYWKLEKNERKERN
LPSNMLPQIYEELVEVVERRGGQQRMLTAAGGSGSNPNTSYVVERPITDVQPSVLPPLLQHQVS
AAPTPALPLPPPCPPPSQQPQAQPPPPIQFPEPFPTLVLIGLVPHFHVLEMCDSSDSDTSEYSNSPA
KRRRRGGGGGEGTSGGTASASTSHEVGTAISKSASIIAEAIQACDEREERRHRDLLSLHERRLKI
EESKTEINRQGISGLVDAINKLANSILALASHKNQSSGPK

>PtrGT44

MGENKGETTKKQQQQQLSSSTSPSPKGPIEESTELRSLHHQQTTSAVVTGAPFIPAPLFVPIGATS
SPFEQHFETVNPKRARYSSAQWKLLPSASQQQQPPTQAQMATVVSSEATPSLITNPTQQKQPRT
TAEASSSDTNSSPLRSPVPSLSAASAQETSKPEGEPHQHQFRKGKYVSPVWKPNEMLWLARAW
RFQYQGGSDLTSRVEQLPETTTGQVTSGDTAIAVQSTRGKTRADKDREVAEFLNRHDVHRDAK
TAGTKWDNMLGEFRKVYEWERGDKREQMGKSYFRLSPYERKAHRLPASFDEEVFEELSQFM
GPKMRTQSRGPSLSGSGDDSSRGTSLAAARALPPPPPFKEDDLSLSGRTRQLVLTSGGEAYFHG
TRGSLLGFDTAVDAAAGSSSSTKELRRIGKVRMTWEESVSLWGEEGEHHRGRVRLQGSSFLNA
DELTFFDDSMVACTMEAFEDGALKGFSVDKFVSGQQIKVFGRRKPSPSASVTPSGFTERVQLPL
TEPSIRLPPWEFQDPTEYYVGCLRVPPTTLPSLFELSWYLQEPPPEELRFPLRRDVYRDLPQGKE
LFFTISNDPLDCRGITYDILSSIIRTNPSISGATSSSRDSFIGVWDDCINRIVSKFCSVEVVIVRKQPS
SPMVDTLQDEWPNVTGFVRNFCLWRGEETDQLREGQVDPSSSIIEKLLWTYLDLPYILGYYAV
GYSVTFCALCRSQDRIIRTDLYSLDLSSPVERLKALVPCYRVAGLLPLLADRCFNNFNNGGTYK
QLAFSDFERVDMGNGNIMEMTPNTVTRLFSSRRKWAAVKEIYDCLDHRIPHAEFIYRSSEKDLA
LVFKPRGLKFKPNNCEQLVEALKYVTQALVALHDLSFMHRDLSWDKVMRRSDSENEWFLSGF
DDAVGAPQLNPVHGIGEARGRHAPEMGRGMHGVKVDVWGVGHLVRTCGLGPNGVPKILREL
QNRCLDQNPEQRPTAADCYHHLLQVQSSLQSSSPGVPY

>PtrGT45

MFEGVPEQFHQFIASRTTSLPPPGLSFPLLQGSSNTTNFPSFYPYTTSSQQVPLQPNLLHPILHQG
APTNKDGEKQENNLVAMNMDQFERERSMPELVNPWSNDEVLALLRIRSSMETWFPEFTWEHV
SRKLAEAGFKRSAEKCKEKFEEESRYFNISNINYNKNYRASFSELEEIYHGDQNPHQEVTAGEK
NKKIDKPGTEDEEQDKMRQNLEEETRIDQTVGNQTDQDNNGKLALVEKSKSNKRKREKKFE
MFKRLCEDIVNTMMAQQEEMHNKLLEDIVKRDEEKVAKEEARKKLEMDRINKELELRAHEQ
ALANDRQATLIRFLKKFTSSDSSVEILGEKIAPDPVNVPNSSNASSSSSLVLEQNPNPASQTSNES
QLKTATSSATALDHQKSTSILAKTNTSWTENQEPQNPTSTLARNIPQVPTTSSTLAVPAAPQNPN
FLNSHSSTSGPPNTLPSHNKVQAKSTYKDDIGKRWPRDEVLALINLRCSLYNNNEDKEGSARA
PLWERISQGMLESGYKRSAKRCKEKWENINKYFRKTKDVNKKRSIDSRTCPYFHQLSTLYNQG
TLVAPDNRSASPENRSSLPETRHSSSSQNGTSNSTLHVGDGEKNMVQRVTCEDGGVAAGMYCF
ASHENAGTELELL

>PtrGT46
MSTPPNTGSPSPPQDLTPLQITALQALPAPPTQPSTNPRRLPPPCWTPDETVALIDAYRDKWYTL
RRGNLKANHWQEVADAVARRCPAASPPKTAVQCRHKMEKLRKRYRTEIQRARSMPVSRFASS
WVHFKRMDGMEKGPQAKADYNSESEENYRTAGNVMNTRSIQKLYRNGMGNPGTGSGNNGL
SGGNSSSGNAGGFRIRIPTGVSIAQPGPKFYAKPEQKYGGGPSMGVNASPNPNLKPNYGGMGS
SSRVMRGGEEMGKKREREPMEELVAAIKVLGDGFVRMEQMKMEMAREMETMRMEMEMKR
TEMILESQQRIVEAFAKALSEKKKRPKRMPSPES
>PtrGT47
MAAAFSALSISPYTLVCRHNSTKHSISCSTASPTTTTIGSRGTKAPPRHKRSERMEGAGKSMEDS
VKRKMEQFYEGSDGPPLRIVPIGGLGEIGMNCMLVGNFDRYILIDAGVMFPDYDELGVQKIIPD
TTFIRRWRHKIEAVIITHGHEDHIGALPWVIPALDHHTPIYASSFTMELIKKRLKENGIFVPSRLK
VFKTKRKFAAGPFEIEPIRVTHSIPDCCGLVLRCADGTILHTGDWKIDESPLDGKKFDRETLEEL
SKEGVTLMMSDSTNILSPGRTISESVVADALLRRISAAKGRIITTQFASNIHRLGSVKAAADLTG
RKMVFVGMSLRTYLDAAWKDGKAPIDPSTLVKVEDIDAYAPKDLLIVTTGSQAEPRAALNLAS
YGSSHALKLNKEDVILYSAKVIPGNESRVMKMMNRISEIGSTIVIGKNELLHTSGHGYRGELEE
VLKIVKPQHFLPIHGELLFLKEHELLGKSTGIQHTTVIKNGEMLGVSHLRNRRVLSNGFVFLGK
ENLQLMYNDGDKAFGTSTELCVDERMRIATDGIVVVSMEILRPQNADGLVENSLKGKIKITTR
CLWLDKGKLLDALHKAAHAALSSCPVNCPLAHMERTVSEVLRKMVRKYSGKRPEVIAVAME
NPAAVLSDELNAKLSGNSHVGLGISALRKMADGHKKKIRVDRKQPDGNGYANLEKTSTQNSE
VDGFEFERELSKEEETSSSPSLAEGHSSDSENQDDFRKSFIPPSPVNELVKSDEDLVPPWEHVNE
LKEDGTISSDDDSLENQNSRSKGSRPVKRNKWKPEEVKSLIKMRGELHSRFQVVRGRMALWE
EISTNLMADGINHSPGQCKYLWTSLAKKYEESKSDKKSQKSWSYFEDMDNILSDSETMATK
>PtrGT48
MVSESNDGQETQELSRPNGVRKEQALDVDDKTKTRHPRWTRQETFVLIESKKVVENRFQWSR
RSTSALGSDQIESKWDSVSSYCSQHGVNRGPVQCRKRWGNMLCDFKKIKTWESQQMNETESF
WMMRNELRRERKLPSFFDKEVYYVLDGRVITTDAIPLSQITFKKQMDCIDRDEAATAEEEEEE
HEYTEANLDSSQCARAEDGTTKKQNLGTKTRKGSMSREGKKRRLSSDESEDPDLEDRLIKVL
GRNMSMLNSQLKAQNINCQLDREQRKDHNDSLVRALNKLTDAIVKIADKL
>PtrGT49
MDLLITGDRQIQNPNDFPLQITPFPPTGNFTIPTSAAAPSAAEAGSSEAASLPPQKLRPIRRTGRV
PSSSREDGEESLRGLVSGQPSEVAAGGGGGSGGGLFDDEFSLCSSDGNDDDLSVGVGESVGRK
RKRKSKIEKFLESLVMKVMEKQEEMHKQLVEMIESRERERVIRDEAWKQQEIERMKRDSEAR
AQETSRNLALISFIQNMTSGHVIEVPQPSMTFSHMVNDGGNADAPIQNDFMGDQSNQRWPEAE
VQALIMLQTAWEQQSRVTGSKGTNIWDAISAGMYNMGYNRTAKKCKEKWENINKHFKMSLG
TAPKKPFQNSTVSPYFPELDTLYNDDFVNLGNGSANTDNQANCALGKG
>PtrGT50
MGSGMLGLELPLHQPENPQNPNHMNHPQVLAYTDHESDHRQHSQQSVKHAYPYASSTKPKQ
LLSPLSDEDEPSFTGDDNTTDGKRKMSPWQRMKWTDHMVRLLIMAVFYIGDDVGSEGSDPIG
KKKPGGLAQKKGKWKSVSRAMMEKGFYVSPQQCEDKFNDLNKRYKRVNDILGKGTACRVV
ENQNLLDTMDLSPKMKEEVRKLLNSKHLFFREMCAYHNSCGHAPSWVAFGSNHSPEMATGPS
PAHAQQHQRCSHSTENAQVVTNSRTETEGSKMAKRAINEEDEVEDDDDDDSDNDEDDCEDD
GDEAIEGNSRHQNGHGQGDEDNRDEKSSRKRPRKDVFSATSPLMQQLNGEIMNVLQDVAKSS
WEKKHWMRLRLMQLEEQQVSYQCQAYELEKQRLKWVKFSSKKEREMEGVKLENERRRLEN
ERMVLIVRKKELELLDTQHQQQQQQQQLPSNKRSDTSSIAG

>PtrGT51
MEGHLHHQHHNQHHLQQYQLLQQQHHISVNVEASDKFPQWSIQETKEFLMIRAELDPTFMEK
KRNKVLWEVISNKMKEKGYNRSAEQCKCKWKNLFTRYKGCETIEPEAMRQQFPFYNELQAIF
TSRMQRMLRAEAEGAATGLKKKAVKLSSDDEDKNEEIIEGEKGGSRKKIKKGKAIGGTSSSGN
SISLKESLEDFTKQQIQMEMQWREVFEARENERMMKEMEWRQTMKQLENDRIVMERRWRER
EEQRRVREEARAEKRDALITALLNKLRREDM
>PtrGT52
MEATQSITAINNKKRKKGNQDCKNSNSSRVLSTPRTRSQVSPEWTAKQALILVNEIAAVEKDCS
KAVSTNQKWKIIVGNCVALGVTHTLSQCRSKWNSLVIEYNQIKKWDKESESRSDFYWSLGCER
RKEFGLPENFDDELFKAIDDYMWSQKEQLDTDPDTDLQKADLLDVIANLERYVEENHQTCCT
KEKPQTIPAEEELHEIQVKEKPQKRLRKEKPQIGNGDEKPKIYSGRKKMPSTEDMEQMMVEKL
SENAEMIQAVVNGNLPEMADLEAADSNNIEGFKTDLIRSQGDKLIACLENIVNTMRQFPWLLQ
DATS
>PtrGT53
MAGFFENTVKKVMDHQEMLHRKFLEVIERMDRERTDREETWRHQEAEKHNREAISRAHERA
STSSREAQIVTYKERITGQSINLPIRMAPPLLQPEISNEPIKEITSTKSDSHSRWPKDEVEALIKVR
SRIEIKFQEPGVKGPLWEEVSSLMSSMGYQRSAKRCKEKWENINKYFRKAKESPERRSQRSKT
CSYFNQLDQLYSRSLINYPSSTTYMPSRGIEFDIEKQGQPEVLEAFAVGKDHLATITNPPGENIKG
AEMGSSRFEFDGIADKNEELEEGSSSRIKKFTKMISKRMANKEKMTVVVTKKMKIEARA
>PtrGT54
MQPSNGGLSYIHHHHHQQQERQQHSMEDDCCCSPSVLQNLNHPCLSHLQQEQHKQPHQVFL
QQHHQLFYPFQQQQQQEQQSQGQGTPQNPSLFTMSFKLGRNEGSGNRKASALNNQDEVAATP
TLLDGNEHINPPHAILMPRSWHPLEDYTTIKEPFWKQLEKKRWGSEEEEISGNRTRYFRPLEIEH
VVDESRERCRNLDIKFPIFNELEAIYSLAKIAEANQTGSGSVLTGDHSPTNAGVSVPSSATNRQN
VGANGAANNVTGVDHGSENSIGEEASLRKSQKRKRKRKLKEKLSYMAGFFENTVKKVMDHQ
EMLHRKFLEVIERMDRERTDREETWRHQEAEKHNREAISRAHERASTSNREAQMVSYIERITG
QSINLPIRMAPPLLQPEISNEPIKEITSTKSDSHSRWPKDEVEALIKVRSRIEIKFQEPGVKGPLWE
EVSSLMSSMGYQRSAKRCKEKWENINKYFRKAKESPERRSQRSKTCSYFNQLDQLYSSSLINY
PSSTTYMPSRGIEFDIEKQGHSEVLEAFAVGKDHLATITNPPGENIKGAEMSSSRFEFDGIAEKNE
ELEEGSSEQDKEVYKDDKQENGEQGKDDGSGDEENEN
>PtrGT55
MDYIEDDPRYPPKSYSLNRSKKHPLYPQRQTPTLHYNYDYDYDYDYLDVDEDGDELDDEFLD
KNDSSYNPGGGYARKFQRNEEAFERYPKRQRLKNPVSNYEFAPRNPRLLAYGDGNSAVEWSE
HEKFVLLEVWGDKFLQLGRNSLRSEDWVDVAEKVSETSKIERNEAQCRQMMDVLKRRYKKE
KAKGGNFSKWAYFNKMDMLMKQESGTVGGFSLACGVDSGEYVFMDTRVYLDRANMNDEM
RDSPCESEEEEEEEEEGGGSAGNDGMKGLRVLAESVQKFGEIYGKIESSKREQMMELERMRIEF
QMDLELQKKQILDRAQAEIAKIREEDDDEEDEDNSDDDDVSGGNISK
>PtrGT56
MEASTTFLENSSAAAGDWEDEEGDEGMRVQAEEGVQCSTANRWPKQETLALLEIRSDMDVAF
RDSVVKAPLWEEVSRKLNELGYNRSAKKCKEKFENIYKYHRRTKGSQSGRPNGKTYRFFEQL
QALDKTNALVSPTSSDKDHCLMPSASVIPVSFIPNDVPCSVQSPRMNCTDATSTSTASTSSEESE
GTRKKKRRLTDFFERLMKEVIEKQENLQNKFLEAIEKCEQERIAREEVWKMQELDRIKREQEL
LVHERAIAAAKDAAVLAFLQKFSEQGIPVQLPDNPTVPMKFPDNQTSPALLSKNQAVPVENVV
KTHENSSVESFVNMSSSRWPKEEIESLIKIRTYLEFQYQENGPKGPLWEEISTSMKNLGYDRSA

KRCKEKWENMNKYFKRVKDSNKKRPGDSKTCPYFQQLDALYREKTRRVDNPSYELKPEELL
MHMMGGQEDQQLPDSATTEDRESENVDQIQVDYRGKEDGDGYGIVAIDPSSLEIME
